# Widespread RNA binding by chromatin-associated proteins

**DOI:** 10.1186/s13059-016-0878-3

**Published:** 2016-02-16

**Authors:** David G Hendrickson, David R. Kelley, Danielle Tenen, Bradley Bernstein, John L. Rinn

**Affiliations:** Department of Stem Cell and Regenerative Biology, Harvard University, Cambridge, MA 02138 USA; Broad Institute of Harvard and MIT, Cambridge, MA 02142 USA; Beth Israel Deaconess Medical Center, Boston, MA 02215 USA

**Keywords:** RNA, lncRNA, RNA-protein interaction, RIP-seq, Chromatin

## Abstract

**Background:**

Recent evidence suggests that RNA interaction can regulate the activity and localization of chromatin-associated proteins. However, it is unknown if these observations are specialized instances for a few key RNAs and chromatin factors in specific contexts, or a general mechanism underlying the establishment of chromatin state and regulation of gene expression.

**Results:**

Here, we perform formaldehyde RNA immunoprecipitation (fRIP-Seq) to survey the RNA associated with a panel of 24 chromatin regulators and traditional RNA binding proteins. For each protein that reproducibly bound measurable quantities of bulk RNA (90 % of the panel), we detect enrichment for hundreds to thousands of both noncoding and mRNA transcripts.

**Conclusion:**

For each protein, we find that the enriched sets of RNAs share distinct biochemical, functional, and chromatin properties. Thus, these data provide evidence for widespread specific and relevant RNA association across diverse classes of chromatin-modifying complexes.

**Electronic supplementary material:**

The online version of this article (doi:10.1186/s13059-016-0878-3) contains supplementary material, which is available to authorized users.

## Background

Control of gene expression is mediated by transcriptional and post-transcriptional mechanisms. Standard models hold that DNA binding proteins (e.g., transcription factors) respond to sequence composition and chromatin context to promote transcription of RNA molecules [11, 17, 58, 62]. Subsequently, a diverse cast of RNA binding proteins (RBPs) binds the nascent transcripts to dictate splicing, stability, localization and translation [19, 26, 33, 35, 59, 83]. Recent advances in systematic profiling of nucleic acid–protein interactions have blurred these conventions, finding that many DNA binding proteins associate with RNA to modulate both transcriptional and post-transcriptional outcomes [1, 7, 15, 28, 39, 44, 60, 71, 89]. Collectively, these studies suggest a more intertwined regulatory network than previously appreciated.

RNA’s role in chromatin formation has long been studied [2, 63]. Recent work has focused on better understanding RNA interactions with chromatin proteins. It has been suggested that a large class of newly discovered long noncoding RNAs (lncRNAs) have functional roles in binding and modulating the activity of proteins involved in chromatin modification [38, 45, 57, 70–72, 82, 84]. For example, the lncRNA Xist plays an integral role in the inactivation of one X chromosome in female mammalian cells by recruiting a variety of transcriptional and epigenetic repressors [10, 54, 55, 65, 76, 89]. Despite the established influence of chromatin on the gene expression changes of development and disease, our current understanding of how chromatin modifications are executed by the cell is incomplete. Though much of the machinery has been detected and biochemical mechanisms described, where and when specific chromatin modifiers take action is unclear. If Xist and other examples are to be generalized, RNA may be an important missing component of these incomplete models of chromatin dynamics.

Multiple groups recently mapped the full spectrum of RNA interactions of one of Xist’s silencing partners, PRC2 [31, 39, 89]. Complementing these data with biochemical assays, they suggest that PRC2 binds numerous transcripts with high affinity but lower specificity than traditionally studied RBPs [12, 13, 31]. This promiscuous binding challenged models purporting lncRNA guidance of PRC2 to specific loci and led to revised models based on PRC2 sensing the presence of RNA, which modulates its activity and or localization [8, 30, 31]. How these interactions and properties extend beyond PRC2 to the many other chromatin-associated complexes remains unknown.

Here, we address this question by surveying RNA interactions of 24 proteins using the same cell type (K562) and cross-linking conditions. Our set includes both traditional RBPs and chromatin-associated proteins (CAPs) that lack classically defined RNA binding domains. We refined a formaldehyde cross-linking RNA immunoprecipitation technique followed by deep sequencing (fRIP-Seq) to perform triplicate experiments that showed high concordance, exceeding previous genome-wide surveys of individual CAPs. We detected widespread binding of CAPs to both lncRNAs and mRNAs, driven by a mix of gene structure and sequence composition preferences. We uncovered many intriguing examples of RNA binding relating to the local chromatin, suggesting that RNA indeed plays important roles in creating and/or maintaining chromatin states. Our data provide a powerful, novel resource towards further dissecting the interplay of RNA and epigenetic regulation across diverse chromatin regulatory complexes.

## Results

### fRIP-Seq: a method for capturing and identifying RNA–CAP interactions

To survey a broad panel of RNA–CAP interactions, we required an immunoprecipitation (IP) method optimized for maximal RNA and protein recovery that is specific, scalable, quantitative, reproducible, and similar to chromatin immunoprecipitation (ChIP) conditions known to readily isolate CAP complexes and recover DNA–CAP interactions. Existing cross-link IP (CLIP) methods for measuring direct RNA–protein binding require large amounts of input RNA, scale poorly for survey purposes across multiple antibodies, and are challenging to assess quantitatively [18, 40, 42, 68].

To address these specific needs, we modified existing RNA IP (RIP) and ChIP protocols that employ formaldehyde cross-linking to prevent post-lysis re-association or “mixing” of RNA-protein complexes similar to RIPiT-Seq [34, 67–69, 77, 78]. We first observed that the percentage of formaldehyde used for cross-linking had dramatic effects on both protein and RNA recovery (Fig. S1 in Additional file 1). High formaldehyde concentrations used for cross-linking resulted in much lower protein and RNA recovery in comparison with lower formaldehyde concentrations. We hypothesized that higher formaldehyde concentrations over-cross-link proteins and nucleic acids into macro-aggregates that either are lost to the insoluble fraction or are too large for effective capture. Indeed, in testing a range of formaldehyde percentages using HNRNPU (a nuclear protein), we found that tenfold lower (0.1 %) formaldehyde cross-linking allowed for considerably more efficient recovery of total RNA, protein, and protein-associated RNA (Fig. S1 in Additional file 1).

**Fig. 1:**
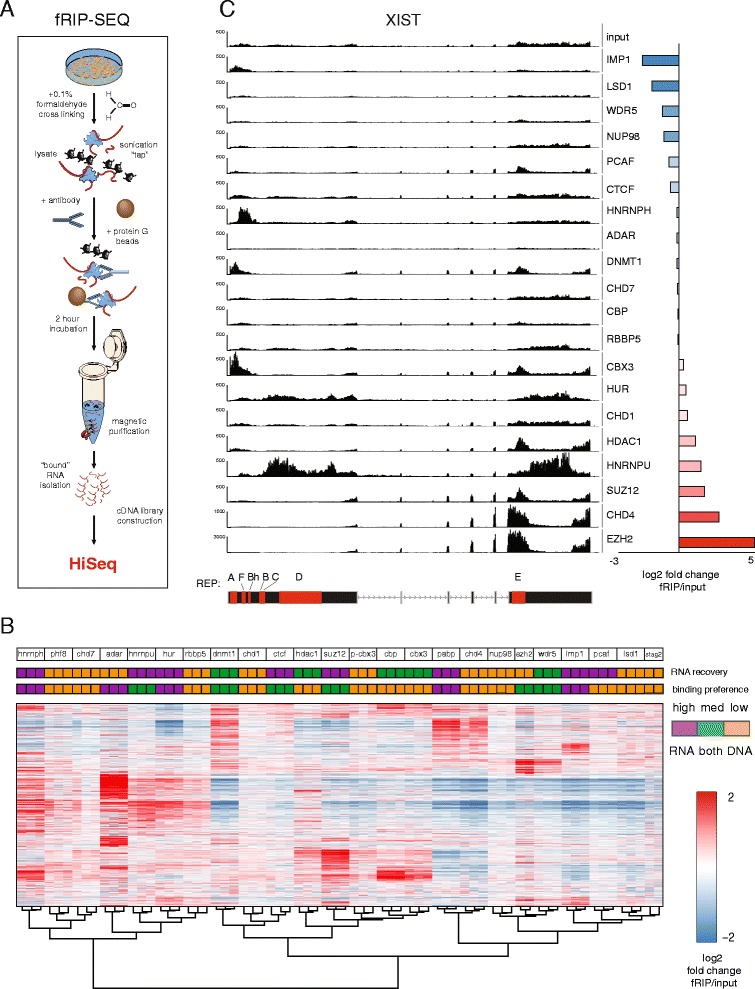
fRIP-Seq reveals widespread binding of chromatin-associated proteins to mRNAs and lncRNAs. **a** Formaldehyde cross-linking RNA–protein complexes enables identification of target RNAs by high-throughput sequencing. **b** We mapped RNA interaction partners for 24 proteins in triplicate and performed hierarchical clustering of log2 fRIP/input fold change over the ~25,000 genes present in at least one condition. Replicates cluster together for every protein. Binding patterns vary between proteins. Neither total RNA recovery from fRIP-Seq (*orange* 1–10 nanogram range, *green* 10–50 nanogram range, *purple* 50+ nanograms) or published nucleic acid binding properties (*orange* DNA, *green* both DNA and RNA, *purple* RNA) can explain the observed clustering solution. **c** The lncRNA Xist is significantly bound by HNRNPU and PRC2 components SUZ12 and EZH2 in our data, validating these known interactions. fRIP-Seq coverage suggests potential binding patterns along the transcript. The coverage scales (y-axis) have maximum coverage 500, except for CHD4 and EZH2, which have maximum coverage 1500 and 3000, respectively

After cross-linking, a 90 second sonication was sufficient for nuclear lysis and chromatin shearing, but gentle enough to lightly fragment RNAs. Following incubation with a targeted antibody, we isolated bound proteins with magnetic beads and purified associated RNA (Fig. S1 in Additional file 1). For the purposes of this study, we refer to this optimized protocol as formaldehyde RNA immunoprecipitation (fRIP; Fig. 1a).

To confirm that 0.1 % formaldehyde cross-linking is sufficient to prevent post-lysis mixing, we queried the association of HNRNPU with cytoplasmic transcripts. We established sets of nuclear and cytoplasmic transcripts as those that were significantly differentially expressed in a comparison of RNA-Seq of nuclear lysate and whole cell lysate. Under native conditions (no cross-linking), HNRNPU enriches for cytoplasmically localized transcripts, suggesting that HNRNPU interacts with these RNAs after cell lysis (Fig. S2 in Additional file 1). However, 0.1 % formaldehyde cross-linking abolishes this association of cytoplasmic transcripts with HNRNPU (Fig. S2 in Additional file 1). Thus, light cross-linking maintains the absence of post-lysis reassociation of RNPs.

After devising and testing the optimized protocol, we compiled a diverse fRIP candidate list (Additional file 2). We included traditional RBPs in our panel as positive controls for known RNA binding preferences and a point of comparison for RNA–CAP binding properties. In addition, recent observations of interaction between chromatin modification and RNA processing suggest that many RBPs may also associate with and influence chromatin [3]. We systematically tested candidate antibodies in fRIP conditions for specific enrichment of the target protein using western blot analysis (Fig. S3 in Additional file 1). From the original candidate list of 36, we were able to cleanly isolate 25 proteins (69 %). Of 25 validated antibodies, 23 reproducibly enriched bulk RNA relative to a negative IgG control (Fig. S1 in Additional file 1).

We performed fRIP on the 23 candidates that had both specific IP and enrichment for RNA interactions. In addition, we included one protein (STAG2, a cohesin subunit) that did not appear to enrich RNA above background as a negative control for background binding of RNA to protein. We also included one antibody that appeared to cross-react with many proteins (SETD2) as a negative control example of a non-specific antibody. To identify the captured RNA associated with our total panel of 25 fRIP experiments, we performed high throughput RNA sequencing (RNA-Seq) on the protein-associated RNA alongside input RNA collected from lysate.

### fRIP-Seq reliably and reproducibly detects widespread binding of CAPs to RNA

Each fRIP-Seq was conducted in biological triplicate (different dates and lysates; STAG2, EZH2, and SETD2 were performed as duplicates) in K562 cells, allowing a thorough assessment of the reproducibility of the experiments. Replicates exhibited remarkable consistency, demonstrated by hierarchical clustering of log2 fold changes of fRIP-Seq over input RNA-Seq (Fig. 1b). For every protein, replicates clustered together.

Further, these data recapitulated known RNA–protein interactions. For example, we observed specific binding of ADAR to Alu sequences, for which they have a well-documented affinity (Fig. S4 in Additional file 1) [49]. Previous CLIP-Seq studies for five RBPs (HNRNPU, CTCF, HUR, IMP1, and HNRNPH1) broadly agreed with our results [22, 32, 40, 48, 61, 74, 86]. Transcripts containing CLIP-Seq peaks showed greater evidence of fRIP-Seq binding than those without, despite all CLIP experiments having been performed in different cell types (Fig. S5 in Additional file 1). SUZ12 and HNRNPU fRIP-Seq experiments clearly detected (>3-fold) established interactions with the lncRNA XIST (Fig. 1c) [14, 25, 53, 90]. Surprisingly, we also found that the ATPase helicase chromatin-remodeling enzyme CHD4 bound XIST >7-fold over input, suggesting that CHD4 is a previously unreported XIST binding protein.

We next asked how transcripts bound by fRIP-Seq are affected upon RBP depletion. In publicly available RNA-Seq measuring gene expression after depletion of five of our proteins (HNRNPU, IMP1, HUR, CTCF, and SUZ12) [13, 40], fRIP-Seq and depletion/control fold changes were significantly correlated (Fig. S6 in Additional file 1). Transcripts identified as bound by fRIP-Seq by the known transcript stabilizer HUR were significantly downregulated following HUR depletion [40, 48, 61]. Transcripts bound by SUZ12 were similarly affected, suggesting a previously unknown stabilizing role.

We observed slight clustering of sequencing reads over specific regions of RNAs, due to sonication shearing prior to protein–antibody pull down. From this coverage bias, we were able to broadly determine regions of protein interaction, but with lower resolution than would be needed for direct binding site detection. For instance, alignment coverage for PABP, a protein that binds polyadenylated transcript tails, was highest over the 3’ end of transcripts (Fig. S7 in Additional file 1). Alternatively, we found that DNMT1 and SUZ12 tended to associate with the 5’ ends of transcripts (Fig. S7 in Additional file 1). Likewise, we found drastic and intriguing differences in fRIP-Seq coverage over individual transcripts like XIST, for which we observed bimodal 3’ binding of HNRNPU and concomitant enrichment of SUZ12 at the site of HNRNPU depletion (Fig. 1c). Lastly, we found that in addition to binding Alu-containing transcripts, ADAR preferentially binds to Alu elements and adjacent regions within transcripts, even after accounting for multi-mapping reads (Fig. S4 in Additional file 1). Collectively, fRIP-Seq not only detects the RNA transcripts bound by a protein but also traces the spatial geography of the interactions.

Having ascertained the resolution and accuracy of fRIP-Seq in measuring RNA–protein interactions, we examined genome-wide trends across the panel of proteins. We observed that CAPs interact with both coding and noncoding RNAs across a large dynamic range of enrichment. Further, CAPs bind a diversity of transcripts; each CAP and RBP had enrichment and under-representation for unique sets of transcripts (Fig. 1b). Importantly, the unique binding signature for each protein was not found to be a function of the physical amount of RNA isolated with each protein (low ~ 1–10 nanogram range, medium ~ 10–50 nanogram range, high ~ 50+ nanograms), nor specific to its recognized status as a dedicated RNA or DNA binding protein (Fig. 1b).

To further investigate potential biases of the fRIP-Seq enrichment profiles, we asked how they relate to transcript localization in the nucleus or cytoplasm. First, we categorized transcripts as enriched in the nucleus versus cytoplasm by comparing RNA-Seq of nuclear and whole cell lysates (Fig. S2 in Additional file 1). We took the most enriched nuclear transcripts and looked at their enrichment patterns across our fRIP-Seq panel (Fig. S2 in Additional file 1). Although enriched by some nuclear-localized proteins (HNRNPH), we found that nuclear-localized transcripts were not preferentially enriched by CAPs as a class compared with known cytoplasmic proteins like IMP1. These data indicate that the enrichment profiles in Fig. 1c are not simply a reflection of the localization of the targeted protein.

As a final test for the possibility of unnatural interaction with cytoplasmic transcripts, we performed a full fRIP-Seq experiment in nuclear lysate for the nuclear protein DNMT1, an exemplar of many of the CAPs in our panel. Cytoplasmic interactions were inconspicuous; fRIP/input fold changes were highly concordant between the two conditions, clustering together among all sequenced samples (Fig. S8 in Additional file 1). Altogether, we established that fRIP eliminates post-lysis mixing, nuclear transcript localization does not bias fRIP-Seq, and nuclear fRIP-Seq produces similar results.

SETD2 replicates sequenced as a negative control for a non-specific antibody produced discordant binding profiles; the replicates did not cluster together when analyzed with the full dataset. In contrast, the two STAG2 fRIP replicates with low enrichment for bulk RNA reproducibly strongly enriched for a small set of 22 genes on a scale of 10–100-fold. Notably, this includes the STAG2 protein binding to STAG2 mRNA (60-fold enrichment). Prior studies establish a precedent for negative controls unexpectedly binding specific RNA targets [26]. As a set, the significantly enriched transcripts are specific to STAG2 and functionally related by the localization of the encoded proteins to centrosomes, centrioles, and spindles (Fig. S9 in Additional file 1). Together, these observations suggest the validity of this experiment and a potential role for STAG2–RNA interactions in chromosome biology.

### CAPs and RBPs enrich for RNA at various stages of processing

**Fig. 2:**
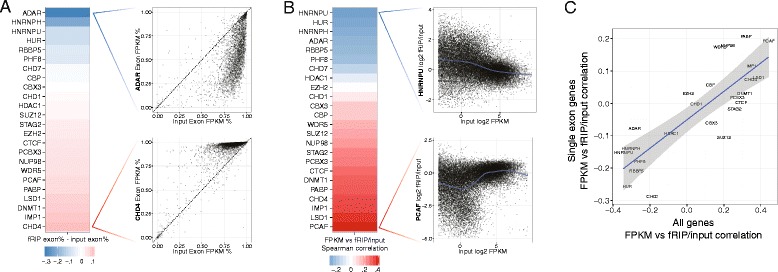
Proteins bind RNA at various stages of RNA processing. The proteins varied on the proportion of intron alignments in the fRIP-Seq versus input. **a** The heat map shows the average proportion of a gene’s FPKM assigned to exon isoforms versus unspliced pre-RNA isoforms (“Materials and methods”). The scatter plots show every gene for ADAR and CHD4. Traditional RBPs ADAR, HNRNPH1, HNRNPU, and HUR likely bind co-transcriptionally; thus, they often immunoprecipitate with unspliced transcripts. **b** RBPs also varied on the degree to which the fRIP/input fold change correlated with input FPKM. The heat map plots the Spearman correlation of these values, and the scatter plots show every gene with a generalized additive model regression line. **c** Relationships between input FPKM and fold change were consistent between single and multi-exonic genes

In studying positional preferences along transcripts, we also observed that fRIP alignments from different proteins varied along a spectrum of the proportion that aligned to introns versus exons. We hypothesized that the proteins bind at different stages during the lifecycle of the RNAs’ post-transcriptional processing. To compare the proteins, we computed the percentage contribution to total gene FPKM (fragments per kilobase per million fragments) by purely exon isoforms versus unspliced pre-RNA isoforms (see “Materials and methods”). In the fRIPs, exonic contribution ranged from proteins that almost exclusively bound exons (CHD4, IMP1, DNMT1, LSD1) to those with far more intron binding (ADAR, HNRNPH1, HNRNPU, HUR) (Fig. 2a). Presumably, exon binders interact with the RNA after transcription and initial processing, while the intron binders are present and bound during transcription. The known roles of intron binders HNRNPU [86], HNRNPH1 [32], and HUR [48] in splicing support their co-transcriptional presence.

The proteins also varied considerably on their preference for binding genes present in the input at low or high abundance, which we assessed by plotting and regressing input FPKM against fRIP/input fold change (Fig. 2b). We noted a relationship between the contribution of intron alignments and the correlation between gene abundance and fold change across proteins (Spearman correlation 0.93; *p* value < 1e-10). The intron binders were more enriched in the fRIP for low abundance genes, particularly those with <10 FPKM (Fig. 2b). In contrast, most other proteins, and particularly the strongest exon binders, preferred higher abundance genes.

We hypothesized that the correlation between intron preference and abundance preference could manifest as a consequence of the co-transcriptional presence of the proteins. For highly abundant genes, usually only a small proportion of RNA for the gene exists at the site of transcription at any given time. If a protein is only binding the gene’s RNA at this locus, it is likely to be depleted for the gene’s overall RNA. Alternatively, a much greater proportion of a transcribed low abundance gene’s RNA would exist at the transcription site. This could more easily lead to enrichment of a protein that binds RNA co-transcriptionally.

To test this hypothesis, and rule out the possibility that the FPKM-dependence of fRIP/input fold change is an artifact of the challenge of estimating abundance from incompletely spliced RNAs, we examined single exon genes. If the same dependence of fold change on FPKM appears for single exon genes, where the challenge of quantifying intron reads is absent, we may proceed with more confidence in the functional relevance of our observations. Indeed, single exon genes demonstrated the same influence of abundance on fRIP/input fold change (Fig. 2c). FPKM versus fold change correlations aligned well for all genes and single exon genes (Spearman correlation 0.80; *p* value < 2.5 e-6).

### CAPs bind to diverse sets of both mRNAs and lncRNAs

**Fig. 3:**
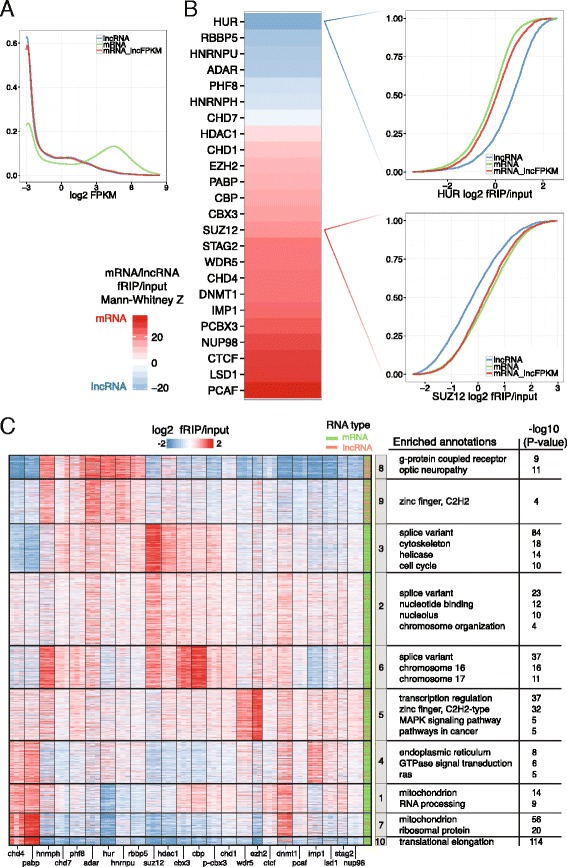
Chromatin-associated proteins bind functionally coherent sets of mRNA. RBPs differed on the degree to which they preferred to bind mRNAs versus lncRNAs. **a** To properly compare the two, we sampled a set of low abundance mRNAs to match the distribution of lncRNAs (referred to as *mRNA_lncFPKM*) and plotted the FPKM distributions for each set. **b** The heat map plots the Z scores of Mann–Whitney U tests comparing the distributions of fold changes for lncRNAs and low abundance mRNAs. To its right, we plot the empirical cumulative distribution functions for HUR and SUZ12. **c** We partitioned significantly enriched genes from all fRIP-Seqs that were also enriched by twofold or more into ten distinct groups using k-medoid clustering. A gene set enrichment analysis using DAVID found significantly enriched functional annotations for each cluster (“Materials and methods”)

Substantial previous work has identified important functional roles for lncRNAs interacting with CAPs [20, 21, 41, 47, 55, 79, 82, 85, 88]. In order to compare and contrast CAP binding of lncRNAs and mRNAs, we first needed to account for the differing abundance levels of these two gene classes. Given our observation of a strong effect of transcript abundance on RBP binding (Fig. 2b) and the paucity of high abundance lncRNAs [5], we sampled a subset of mRNAs to match the lower abundance distribution of lncRNAs (Fig. 3a).

Low abundance mRNA versus lncRNA enrichment spanned a wide range across the panel (Fig. 3b). Surprisingly, we did not find a prevalent bias for lncRNAs over mRNAs amongst our CAPs, but rather a slight (HDAC1, CBX3, SUZ12, WDR5) or even strong preference for mRNAs (LSD1, CTCF, PCAF). In fact, the highest relative lncRNA/mRNA enrichment was observed primarily among the traditional RBPs (HUR, HNRNPU, HNRNPH1 and ADAR, but not IMP1 and PABP).

We next explored the idea that lncRNAs, as potential “guides” for chromatin modifying complexes, might be more selective in their associations with CAPs compared with mRNAs. To determine the selectivity of lncRNAs for CAPs in our panel, we calculated a CAP binding specificity score for each transcript using an entropy-based metric that relies on Jensen-Shannon (JS) divergence (“Materials and methods”) [5]. This specificity metric (ranging from 0 to 1) quantifies the similarity between a transcript’s binding pattern across our panel and a predefined pattern that represents the extreme case in which a transcript associates with only one CAP. By this measure, lncRNAs were significantly more specific in their binding preferences across the CAPs compared with an abundance-matched sampled population of mRNAs (Fig. S1 in Additional file 1). Thus, lncRNAs may interact less promiscuously with CAPs compared with mRNAs.

### CAPs associate with functionally coherent sets of mRNAs

Given that CAPs interact widely with mRNAs, we next asked whether these mRNAs belong to coherent gene expression programs. We took advantage of the fact that, unlike ncRNAs, mRNAs have vast collections of functional annotations. We clustered all genes into ten discrete groups using k-medoid clustering on their fRIP-Seq enrichments to isolate distinct patterns amongst genes and between fRIPs (Fig. 3c; “Materials and methods”). Strong relationships between specific fRIPs (e.g., CHD4 and PABP or CBX3, SUZ12 and CBP) from the hierarchical clustering in Fig. 1 are generally preserved and the enrichment patterns driving the clustering are easily discernible.

We analyzed each cluster for enrichment of a variety of functional annotations using the Database for Annotation, Visualization and Integrated Discovery (DAVID; “Materials and methods”) [27]. We found hundreds of enriched terms and disease associations within the fRIP-Seq clusters (Additional file 3). Clusters 1, 7 and 10 exhibit highly enriched terms and are composed of transcripts primarily associated with CHD4, DNMT1, and PABP. Functional annotations enriched in this cluster are generally related to translation and mitochondria. Another example is the related set of clusters 2 and 3, wherein association with the PRC2 subunit SUZ12 is the dominant pattern. These clusters are strongly enriched for cytoskeleton, microtubule, nucleotide binding, cell cycle terms and alternatively spliced genes. Combinatorial binding was evident; many transcripts bound multiple RBPs and CAPs either simultaneously or at distinct temporal phases in the transcripts’ life cycles. Consistent with our observation that RBPs bind more readily to lncRNAs than CAPs, clusters 8 and 9 are dominated by association with HNRNPH1, HNRNPU, ADAR and HUR and are enriched for lncRNAs.

While the underlying biology driving the observed functional relationships between fRIP-enriched sets of genes is unclear, their existence argues that the interactions captured via fRIP-Seq are nonrandom and that the widespread mRNA-CAP associations may be biologically relevant.

### CAPs specifically associate with a variety of transcript features

**Fig. 4:**
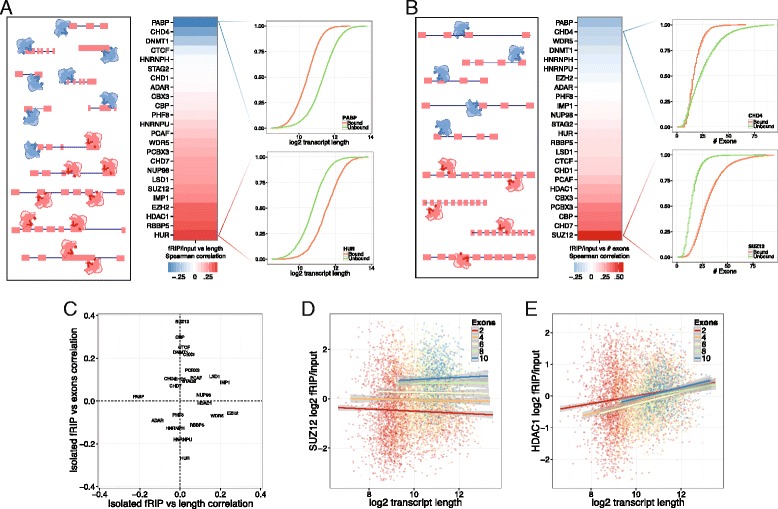
Chromatin-associated proteins prefer specific gene structure properties. The proteins had strong preferences for transcript length (**a**) and exon number (**b**). The heat maps plot the Spearman correlation between fRIP/input fold change and length or exon number. To their right, we plot empirical cumulative density functions for specific proteins exemplifying substantial correlations. **c** Because length and exon number are highly correlated, we isolated the role of each using semipartial correlations. We regressed fold change against exon number and computed Spearman correlation of the residual against length (*Isolated fRIP vs length correlation*) and vice versa (*Isolated fRIP vs exons correlation*). As can be seen in the resulting plot, and further explored in (**d**), SUZ12 is affected by exon number rather than length. **e** In contrast, HDAC1 correlates with length at every level of exon number plotted

We next turned to exploring the RNA properties that determine protein binding. For example, it has been reported that EZH2 has greater in vitro affinity for long RNAs [12, 13]. To assess this attribute for EZH2 and all proteins surveyed by fRIP-Seq, we computed the Spearman correlation of transcript length and fRIP/input fold change over all mRNAs (Fig. 4a). We set gene lengths to the average length of the gene’s isoforms weighted by their input FPKM. In addition to validating the preference of EZH2 for longer transcripts, we discovered that many more CAPs, including RBBP5 and HDAC1, also strongly prefer longer transcripts (Fig. 4a). In contrast, CHD4, DNMT1, and CTCF bound shorter genes.

Recent studies have uncovered a regulatory layer interfacing co-transcriptional RNA splicing and chromatin [3, 43, 44, 51, 52, 56, 60, 73, 91]. Because longer genes tend to have more exons, we wondered whether the length preference of CAPs might be more attributable to the number of exons in the bound transcripts, potentially via interaction with the splicing machinery. Similar to above, we assigned each gene the average exon number of its isoforms, weighted by their input FPKM. Spearman correlations of fRIP/input fold change and exon number matched those for length (Fig. 4b), suggesting that the relationship of length and exon number to binding is confounded.

To differentiate the role of length versus exon number, we computed a semipartial correlation with fRIP/input fold change for each. More specifically, we performed a regression for one attribute to predict fold change and computed the Spearman correlation between the residuals and second attribute. If only one attribute (such as length) truly mattered, the regression for length would model the data completely and no correlation with exon number would remain in the residuals. Comparing these two statistics, we found that numerous proteins that appear to depend on transcript length (SUZ12, CBP, CHD7, PCAF) respond far more to the number of exons (Fig. 4c). For these proteins, length correlation subsides after accounting for the effect of exon number.

SUZ12 exemplifies this exon number preference. We observed that SUZ12 fRIP/input fold change has Spearman correlation 0.37 with exon number after length-normalization, but an insignificant 0.03 correlation with length after exon-normalization. To further demonstrate this property, we observed that a positive correlation between transcript length and SUZ12 fRIP/input fold change was absent among sets of transcripts with an equal number of exons (Fig. 4d), but greater exon numbers increased the average fold change among the genes. In contrast, HDAC1 exemplified another set of proteins for which transcript length appears to be the more important variable; the same slope relating length to fold change appears for genes with every number of exons (Fig. 4e).

In summary, structural properties of the genes affect their binding by CAPs. Though previous work has characterized a preference of PRC2 subunit EZH2 for longer transcripts, we found here that for PRC2 subunit SUZ12, the number of exons in the transcript, rather than its length, is a more dominant determinant of binding.

### CAPs bind to specific sequence motifs

We next asked whether our panel of CAPs and RBPs have sequence composition binding preferences in addition to the gene structure preferences described above. To this end, we performed a search for motifs whose presence in gene transcripts had high mutual information with the transcripts’ fRIP/input fold changes for each protein (“Materials and methods”).

**Fig. 5:**
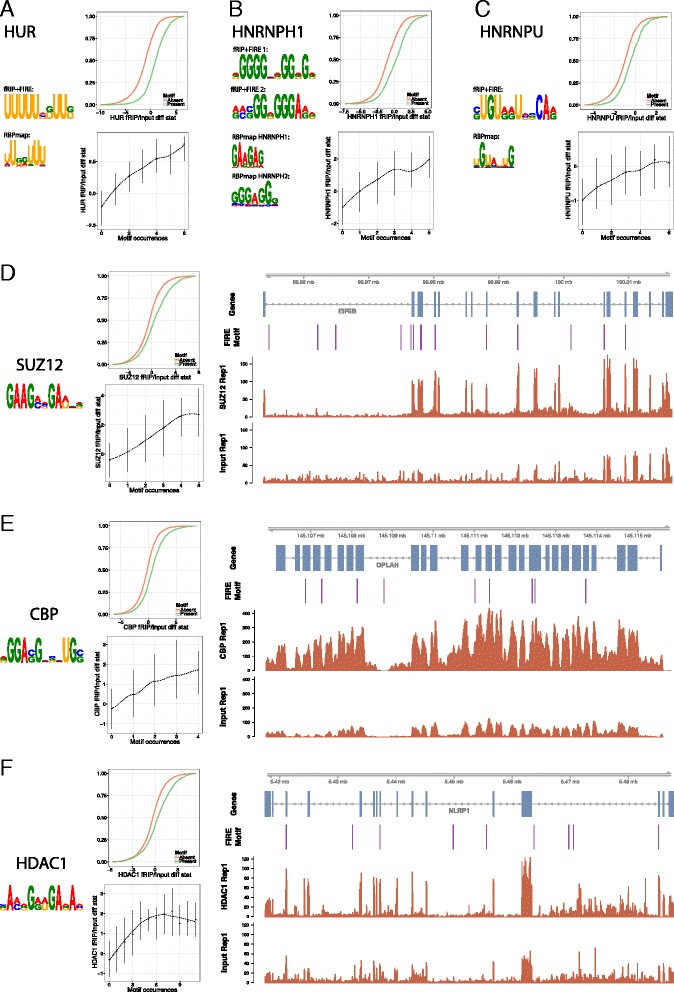
Chromatin-associated proteins prefer specific sequence motifs. We searched for motifs that have high mutual information with the fRIP/input differential expression statistic using FIRE (“Materials and methods”). Motifs discovered for HUR (**a**), HNRNPH1 (**b**), and HNRNPU (**c**) matched well to known motifs in the RBPmap database [64]. For each motif, we plotted the empirical cumulative density function of the fRIP/input statistic for genes with and without the motif. Below that, we plotted the 25th, 50th, and 75th percentiles of the fRIP/input statistic for genes containing the specified number of motif occurrences. We discovered novel motifs at similar levels of significance for CAPs SUZ12 (**d**), CBP (**e**), and HDAC1 (**f**)

Even though the fRIP-Seq protocol does not include shearing RNA down to binding site resolution, we discovered many significant motifs in transcript-wide searches. The sequence binding preferences of traditional RBPs HUR, HNRNPH1, and HNRNPU have been previously explored, and we recapitulated those preferences here with U-rich motifs for HUR [40, 48, 61] (Fig. 5a), AG-rich motifs for HNRNPH1 [6, 23] (Fig. 5b), and a UGU motif for HNRNPU [29, 86] (Fig. 5c).

Having established that fRIP-Seq can find known binding motifs, we turned to the CAPs, for which knowledge of sequence binding preferences is sparse. As with the traditional RBPs, we found many motifs for the CAPs, which were significant at similarly high levels. SUZ12 had affinity for the motif GAAGMHGAW and other AG-rich motifs, exemplified by the EIF5B locus (Fig. 5d). Supporting its strength, transcripts containing three instances of this motif were bound at a fourfold higher level on average. We discovered motifs for CBP and HDAC1 with effects of comparable magnitude (Fig. 5e, f).

DNMT1 was enriched for a GC-rich motif, but only in lncRNAs (Fig. S11 in Additional file 1). Further analysis of this motif uncovered that it was highly biased towards the 5’ end of genes, similarly to DNMT1 coverage overall (Fig. S11 in Additional file 1). Browsing individual genes suggested that the motif often occurs in CpG islands (Fig. S11 in Additional file 1).

Interestingly, many of the proteins responded to similar motifs. The motif UUUUAAAA and slight variations were extremely polarizing to our panel. Seven proteins, including RBBP5 and IMP1 most significantly, bound RNAs containing the motif and did so with greater fold changes per each additional motif occurrence (Fig. S12 in Additional file 1). Alternatively, 15 proteins, including CTCF most significantly, avoided genes containing the motif (Fig. S12 in Additional file 1). Although AU-rich sequences are a well-studied class of post-transcriptional regulatory elements [9], this particular motif has not been a specific focus of these analyses. The motif is highly enriched at the 3’ ends of transcripts, and motif occurrences in 3’ UTRs, introns, and lncRNAs are each more conserved than background sequence of those annotation classes (Fig. S12 in Additional file 1). Though different from the consensus polyadenylation signal (PAS) AAUAAA, we hypothesized a potential relationship between the two. We compared motif occurrences to direct RNA sequencing (DRS) mapping polyadenylation sites in K562 [50], but no obvious patterns emerged (Fig. S12 in Additional file 1). Altogether, these lines of evidence suggest a possible, but presently unclear, functional role for UUUUAAAA in post-transcriptional regulation.

To more fully represent the binding preferences of many related motifs and to measure the overall ability of RNA sequence composition to predict protein binding, we performed a linear regression on k-mer counts to predict the transcripts’ fold changes. The variance explained by binding predictions for unseen transcripts increased with k for nearly all proteins up to a length of k = 7 (Fig. S13 in Additional file 1). The Alu 7-mers for ADAR and G-rich 7-mers for HNRNPH1 drove the highest accuracy predictions of all of the proteins, explaining ~38 % of the variance in log2 fold change. Binding of the traditional RBPs tended to be better predicted by sequence composition, but many CAPs were also modeled well, including RBBP5, CTCF, CBP, and SUZ12. Collectively, our analyses discovered known binding motifs and new trends in noncanonical CAP binding preferences.

Transposable elements (TEs) can serve as a source of sequence motifs with an inherent evolutionary history. Thus, we also asked whether specific classes of TEs in the transcripts affected protein binding. Mentioned above and well-known, ADAR binds Alu elements in both orientations (Fig. S14 in Additional file 1) [49]. We additionally found dozens more significant associations between protein binding and the presence of specific TE families. Transcripts containing antisense Alu elements had greater fold changes in the HUR fRIP, reflecting an interaction recently described in three independent CLIP-Seq datasets with the poly-U stretches of antisense Alu [36]. Though TE preferences within mRNAs and lncRNAs were broadly similar, an association between DNMT1 and sense strand ERV1 was specific to lncRNAs (Fig. S14 in Additional file 1). ERV1 insertions appear to have played a role in the origin of many lncRNAs [37].

Between motif searches, k-mers, and TEs, we detected a variety of known and novel sequence binding preferences of the proteins analyzed, including initial evidence that even CAPs lacking traditional RNA binding domains have greater affinity for certain sequence motifs.

### CAP binding relates to local chromatin

To explore the relationship between CAP binding to RNAs and the local chromatin of the bound RNAs’ loci, we compared fRIP-Seq with all ENCODE ChIP-Seq and reduced representation bisulfite sequencing (RRBS) mapped in K562. Because some chromatin marks are more relevant in either the promoter or spanning body of genes, we computed promoter-based and gene body-based statistics to measure the magnitude of binding for each mark and gene (see “Materials and methods”).

First, we asked whether CAPs bind RNA from loci where they concurrently bind DNA, perhaps because the proteins bind the RNA due to its proximity. We examined the Spearman correlations across all genes between fRIP-Seq and ChIP-Seq for 11 proteins with both data types (Fig. S15 in Additional file 1). Coordinated DNA and RNA binding is not apparent, suggesting that other factors are more important to determine RNA binding and that DNA occupancy alone is insufficient to drive association with transcripts in close proximity.

**Fig. 6:**
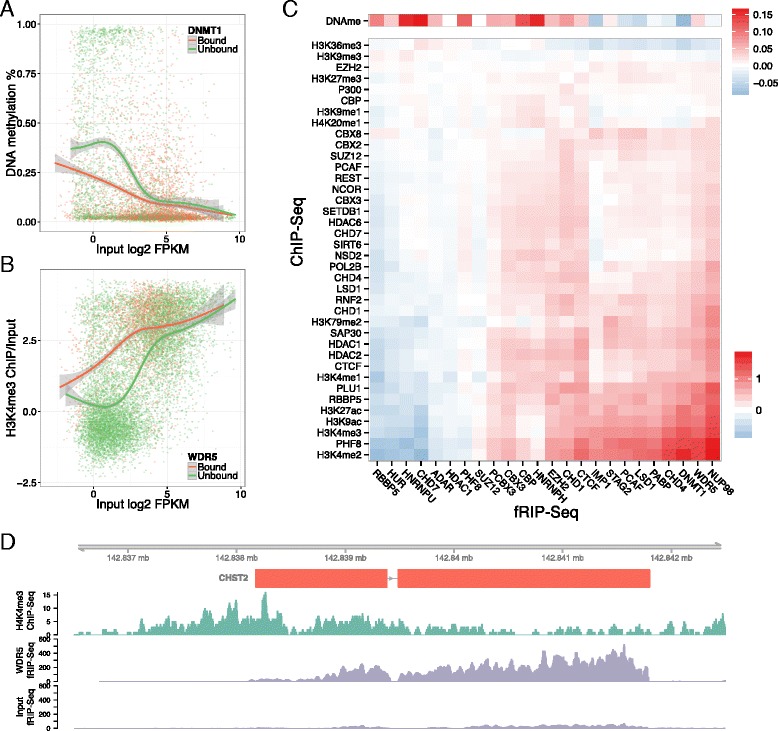
Protein binding to RNA relates to local chromatin. Because chromatin marks measured by RRBS (DNA methylation) or ChIP-Seq (histone modifications and modifiers) correlate with gene abundance (Fig. S16 in Additional file 1), we plotted this relationship separately for genes bound and unbound by each protein. **a** DNMT1-bound RNAs have less DNA methylation in their promoter, shown as a scatter plot of every gene with a generalized additive model regression. **b** WDR5-bound RNAs have more H3K4me3 in their promoter. **c** For each chromatin mark and protein, we plotted the difference between the bound and unbound regression lines as a heat map (“Materials and methods”), revealing a clear difference in the relationship of certain proteins to activating chromatin marks. **d** CHST2 exemplifies a WDR5-bound RNA with ample H3K4me3

Extending to all ChIP datasets, since chromatin marks correlate very strongly with gene expression (Fig. S16 in Additional file 1), raw correlations between fRIP/input fold change and promoter or body-based ChIP were confounded with the tendency of the proteins to bind lower or higher abundance transcripts (Fig. S17 in Additional file 1); that is, proteins that bound higher abundance genes positively correlated with active chromatin marks and vice versa. However, many intriguing relationships appear when plotting the ChIP statistics for significantly bound and unbound RNAs across input FPKMs. Correlations between chromatin marks and gene abundance emerge and can be normalized for. Matching and generalizing previous analysis of DNMT1 binding to RNA at the CEBPA loci, we observed lower levels of DNA methylation in promoters of genes bound by DNMT1 genome-wide at all abundance levels (Fig. 6a). Interestingly, DNMT1 binding does not appear to affect gene body DNA methylation, but CTCF binding has a strong relationship: CTCF-bound RNAs have higher levels of methylation across the span of the gene (Fig. S18 in Additional file 1). This is consistent with prior work linking CTCF to DNA methylation and splicing [75].

For a more global view, we quantified all fRIP-chromatin mark relationships by measuring the gap between regression lines for bound and unbound RNAs across input FPKM (Fig. 6c; “Materials and methods”). Clustering analysis revealed that chromatin marks group with respect to their relationship with gene activation. RNA binding of many CAPs (e.g., DNMT1, NUP98, WDR5, PCAF, and LSD1) correlated with higher presence of activating modifications like H3K4me3 and H3K27ac. Differences between bound and unbound RNAs were not as apparent for silencing modifications like H3K9me3 and H3K27me3. Greater levels of H3K4me3 in promoters of genes bound by WDR5 (Fig. 6b), as exemplified by CHST2 binding (Fig. 6d), is of particular interest because WDR5 participates in a complex that writes the H3K4me3 mark and has previously been implicated for recruitment by RNA [20, 85, 87].

In summary, the presence of this variety of known and novel relationships suggests a role for RNA–protein interactions influencing the maintenance and dynamics of chromatin states.

## Discussion

Using an optimized and scalable protocol for cataloguing RNA–protein interactions, including those on and around chromatin, we have demonstrated that a diverse set of proteins known to associate with and/or modify chromatin also widely interact with thousands of coding and noncoding RNA transcripts. We recapitulated previously known RNA–protein interactions and found that, like traditional RBPs, CAPs interact with functionally coherent sets of RNAs via specific transcript features in a combinatorial manner. RNA–CAP binding relates to the local chromatin of the RNAs’ loci, adding evidence to support a crucial role for RNA–protein interactions in chromatin modification.

Our fRIP-Seq technique enabled the highly reproducible mapping of a diverse set of RNA targets for 24 proteins. Profiling many proteins in parallel is a powerful method to account for protocol artifact or common background noise arising from promiscuous or highly abundant RNAs that bind protein or magnetic beads indiscriminately [4, 18]. RNA recovery and input library construction (low versus high) did not predict correlation between experiments, ruling them out as confounding factors. Concordance with CLIP-Seq, RBP depletion assays, and individually measured and functionally established interactions further support the validity of our data.

In two cases (SUZ12/EZH2 and WDR5/RBBP5), fRIP-Seq did not map RNA interaction partners of two proteins known to function together in a complex with the concordance one might expect. There are many possible contributing factors for this observation, primarily revolving around the fact that the RNA binding properties of these proteins are poorly understood. The proteins exist in the cell both in and out of the complex, and there is no reason to believe that the proteins in isolation would bind to similar transcripts as each other. If fRIP-Seq is preferentially capturing these RNA interaction partners, then differing profiles would be the expected result.

We observed RNA–protein interactions at various stages of RNA processing, indicated by the quantity of intron alignments in each fRIP-Seq. Known co-transcriptional binders ADAR, HNRNPH1, HNRNPU, and HUR had the most intron alignments. Co-transcriptional binding also led to different patterns relating gene abundance to fRIP/input fold change. These correlations with abundance were recalled and normalized for in downstream analyses, such as comparisons with local chromatin.

Though fRIP-Seq does not pinpoint interaction sites to the same resolution as CLIP-Seq, we nevertheless discovered many binding preferences for the proteins measured using transcript-wide analysis. This included reproducing the known sequence motifs bound by ADAR, HNRNPH1, HUR, and HNRNPU from whole transcript motif searches. Preferred sequence motifs were found for CAPs as well, with similar degrees of evidence as those known motifs. For example, we discovered an AG-rich motif predictive of SUZ12 binding.

In addition to sequence preferences, we found that fRIP-Seq enrichment for many proteins correlated with transcript length and number of exons. In particular, though a PRC2 preference for longer RNA transcripts had previously been observed, we found here that length correlation manifests through a far stronger preference by SUZ12 for transcripts with more exons. Current models for the role of PRC2–RNA interactions posit that PRC2 maintains gene silencing by writing the silencing mark H3K27me3 only in the absence of RNA [8, 30, 31]. Our observations suggest a revised hypothesis whereby obfuscation of PRC2 silencing may further require spliced RNA, sensed by SUZ12 interaction. Given the apparent ubiquitous transcription of the genome, this distinction is an important one, as it would substantially limit the pool of RNA that can modulate PRC2 activity.

Much previous work on RNA binding partners of CAPs has focused on ncRNA. Here, we surprisingly detected substantial binding of CAPs to mRNAs, too. Although we observed weaker enrichments of lncRNAs by CAPs in comparison with mRNAs, we did detect that lncRNAs are more selective and associate with fewer CAPs on average than mRNAs. However, our data overall suggest that lncRNA-CAP binding is not the dominant feature of the RNA–CAP interactome.

Instead, RNA may more generally provide a communication medium between the genome and CAPs. We observed widespread correlations between CAP fRIP-Seq enrichment and local chromatin state. Matching a previous analysis, which suggested that DNMT1 would not methylate DNA in the presence of RNA [15], the promoters of DNMT1-bound RNAs had lower levels of DNA methylation. Furthermore, we discovered a novel relationship between WDR5 binding to RNAs and the H3K4me3 levels of the transcripts’ promoters; loci with bound RNA have more H3K4me3, which could be the result of RNA recruitment of WDR5 and the MLL complex to further solidify an open and active promoter state in a positive feedback loop.

Our analysis leaves open the question of what happens to mRNAs bound by CAPs. One could imagine these interactions are transient light disturbances to the mRNA on its journey to translation. Alternatively, a small proportion of transcribed mRNA copies may be diverted to permanent interaction with CAPs and sequestered away from translation. Follow-up work will be needed to differentiate these outcomes and clarify the role of mRNAs in chromatin modification processes.

## Conclusions

Our introduction of fRIP-Seq and panoramic profiling of RNA interactions with chromatin-associated proteins will enable many future analyses to further dissect the role of RNA in chromatin processes. The dual nucleic acid affinity of CAPs is an intriguing feature that, with further study, may unify the separate paradigms of RNA-mediated chromatin regulation of transcription with chromatin-mediated post-transcriptional regulation of RNA. While we have provided a static snapshot of the cell, the open questions of how chromatin is modified are most relevant to the dynamics of development and disease. The framework applied here provides an important lens with which to study the chromatin regulation of these cell state changes.

## Materials and methods

### Cell culture and cross-linking

K562 cells (ATCC catalog #CCL-243) were grown in RPMI 1640 (Invitrogen, catalog #22400105) with 10 % fetal bovine serum (FBS) and 1 % Antibiotic-Antimycotic 100× (Invitrogen, catalog #15240062). We collected cells with a gentle 5 minute spin at 500 g and washed these with room temperature phosphate-buffered saline (PBS). We re-suspended at 5e6 cells per ml in room temperature RPMI media without FBS or antibiotic-antimycotic and added formaldehyde to a final concentration of 0.1 %. We cross-linked at room temperature for 10 minutes and then halted it by quenching for 5 minutes at room temperature after adding glycine to a final concentration of 125 mM at a medium pace. We spun cells for 5 minutes at 500 g and then washed them twice in 4 °C PBS. We flash froze pellets of 10e6 cells and stored them at −80 °C.

### fRIP

We re-suspended frozen pellets in 1 mL of RIPA lysis buffer (50 mM Tris (pH 8), 150 mM KCl, 0.1 % SDS, 1 % Triton-X, 5 mM EDTA, 0.5 % sodium deoxycholate, 0.5 mM DTT (add fresh) + protease inhibitor cocktail (Thermo Scientific, PI-87785) + 100 U/ml RNaseOUT™ (Life Technologies, 10777–019)). We incubated cells at 4 °C for 10 minutes before lysing on a Branson® digital sonifier using 10 % amplitude for 0.7 seconds on and 1.3 seconds off at 30 second intervals for a total of 90 seconds. We used chilled tube holders and swapped them out between shearing runs to reduce temperature elevation. After lysis, we spun the lysate at 4 °C max speed for 10 minutes. We collected supernatant and diluted by adding equal volume of fRIP binding/wash buffer (150 mM KCl, 25 mM Tris (pH 7.5), 5 mM EDTA, 0.5 % NP-40, 0.5 mM DTT (add fresh), 1× PIC (add fresh), 100 U/mL RNaseOUT (add fresh)). At this point, we removed 50 μl of lysate for input sample and stored at −20 °C for later RNA purification and library construction. After dilution, we clarified the lysate by passage through a 0.45 μM syringe filter. We then “pre-cleared” filtered lysate by incubating with Dynabeads® Protein G (Life Technologies catalog #10004D) at a concentration of 25 μl of beads per 5 million cells for 30 minutes at 4 °C with slow rotation. We flash froze pre-cleared lysate in 1 mL aliquots of ~5 million cells and stored it at −80 °C. For fRIP, we thawed lysate on ice and added 6 μg of *HuR* antibody (Santa Cruz, sc-5483). After addition of antibody, we rotated lysate at 4 °C for 2 hours before adding 50 μl of Dynabeads® Protein G. We rotated beads and lysate at 4 °C for 1 hour before washing twice with 1 mL of fRIP binding/washing buffer + 1× PIC and 100 U/mL RNaseOUT. After the final wash, we removed the supernatant and froze and stored the beads at −20 °C.

### RNA purification and library construction

We re-suspended the frozen beads in 56 μl of RNase-free water and added 33 μL of 3× reverse-crosslinking buffer (3× PBS (without Mg or Ca), 6 % N-lauroyl sarcosine, 30 mM EDTA, 15 mM DTT (add fresh)), 10 μl of Proteinase K (Life Technologies, catalog #AM9516), and 1 μl of RNaseOUT to both the re-suspended beads and input sample. We performed protein degradation and reverse-crosslinking for 1 hour at 42 °C, then another 1 hour at 55 °C. We added beads and reaction buffer to 1 mL of TriZol (Life Technologies, 15596–026). After agitation, we added 200 μl of chloroform followed by ~15 seconds of vigorous agitation and a 20 minute microcentrifuge spin at 4 °C max speed. We collected the aqueous layer, added it to 750 μl of ethanol + 1 μl GlycoBlue™, and ran it over a Qiagen RNeasy® min-elute column (Qiagen, catalog #74204). We extracted RNA using the buffer RWT/3× isopropanol modification detailed in “Appendix B: Optional On-Column DNAse Digestion…” of the *Qiagen miRNeasy® Mini Handbook*. We eluted RNA in 15 μl of RNase-free water. To remove ribosomal RNA, we fed ≥70 ng of input and fRIP RNA into the Ribo-Zero™ Magnetic Gold Kit (Epicentre, catalog #MRZG12324) followed by a cleanup using Agencourt RNAClean XP beads (Beckman Coulter, catalog #A63987) and elution with 19.5 μL of Elute, Prime, Fragment mix from the TruSeq RNA Sample Preparation Kit (Illumina, catalog #RS-122-2001). We performed library construction per the vendor’s instructions, starting with the “Incubate RFP” step. We pooled the resulting cDNA libraries and subjected them to paired-end sequencing on an Illumina HiSeq 2500 at a depth of 31 base pairs per read.

### fRIP-Seq computational analysis

We aligned sequencing reads to human genome assembly hg19 and GENCODE v18 reference annotation [24] using TopHat [81]. We estimated transcript and gene abundances, as well as depletion/ enrichment significance using Cuffdiff 2 [80]. In addition to the standard exon annotation, we estimated abundances on an augmented version of the annotation to which we added an unspliced pre-RNA isoform for every unique isoform start and endpoint. This quantification proved useful in some analyses, such as measuring the contribution of intronic reads from unprocessed transcripts.

### Cluster and functional annotation analysis

We limited cluster analysis in Fig. 1 to genes with expression that was high enough in at least one condition such that Cuffdiff 2 was able to test for enrichment/depletion in at least one fRIP-Seq versus input comparison. For each gene, we added a pseudocount of 1 FPKM before calculating the log2 fold change fRIP/input. We hierarchically clustered these values across genes (rows) and fRIPs (columns) using Pearson correlation distance and Ward’s agglomerative method.

We performed K-medoid clustering (using the R package PAM) on only genes that were called as significantly enriched by Cuffdiff and enriched at greater than twofold over input in at least one replicate. We clustered using k = 10 and Euclidean distance. To order the clusters for visual representation in a heat map (Fig. 3), we performed hierarchical clustering on median log2 fold change for each cluster (row) and each fRIP (columns). To annotate the clusters, we searched for functional terms enriched in each cluster’s genes using DAVID [27].

### Motif analysis

We used FIRE to search for motifs that have high mutual information with fRIP-Seq enrichment [16]. FIRE requests an input dataset consisting of nucleic acid sequences and a statistic assigned to each. For a higher resolution view of fRIP/input enrichment, we created an augmented annotation in which every intron was included as an isoform, extended on both sides to include the adjacent exons. For each protein, we then chose the most expressed isoform for every gene and assigned them the isoforms’ Cuffdiff differential expression test statistic.

Choosing an appropriate seed size for motif searches on full transcripts of varying size is more challenging than the typical application of equally sized promoters. We sought to focus on a middle range of the transcript length distribution so that the chosen seed size was not wildly inappropriate for many transcripts. Accordingly, in an initial analysis we allowed only transcripts whose length is within a factor of sqrt(10) from the distribution median; thus, all included transcripts have length within a factor of 10 of all other transcripts. We then chose the smallest k-mer seed size for which one would expect every k-mer to occur by chance in <1 % of transcripts of that length. Because transcript lengths are log-normally distributed, half of the transcripts are longer and half are shorter than the median transcript length, for which the chosen seed was aimed. For mRNAs, the median transcript length in GENCODE v18 is 1997 nucleotides, suggesting 10-mer seeds. Because this large k-mer size might miss some of the smaller k-mer motifs typical of RBPs [66], we performed additional runs of FIRE using a transcript length distribution chosen to be smaller and more appropriate for an 8-mer-seeded search. Here, we limited transcripts to length between 400 and 4000 nucleotides.

### K-mer analysis

If sequence preferences are driven by more general sequence composition preferences that cannot be so easily represented by regular expression or position weight matrix motif models, then fRIP-Seq enrichment of gene transcripts may be more effectively modeled by considering all k-mers. To this end, we performed a regression to assign weight coefficients to all k-mers for the same input datasets described above. To avoid overfitting, we performed ridge regression, which minimizes not only the distance between model predictions and actual values but also the magnitude of the weights. We chose the alpha parameter that varies the emphasis of these two competing objectives by evaluating fivefold cross-validated mean squared error over a parameter grid. More complex techniques (partial least squares and support vector regression) failed to yield significant gains.

### CLIP-Seq analysis

To assess the concordance between fRIP-Seq and CLIP-Seq, we downloaded six datasets mapping five proteins (HNRNPU [86], CTCF [74], HUR [40, 48, 61], IMP1 [22], and HNRNPH1 [32]). We mapped reads and called peaks using a previously described protocol [36]. We considered a gene to be targeted if an exonic peak was detected.

### ChIP-Seq analysis

We downloaded aligned sequencing reads in BAM format for all K562 ChIP-Seq experiments performed by the ENCODE project from https://www.encodeproject.org.

We assigned every transcript two scores measuring the enrichment of ChIP alignments over input alignments. For the first score, we computed log2 ChIP/input alignments for a promoter region of 3 kb, centered at the transcription start site. For the second, we computed log2 ChIP/input alignments for the entire transcript span. We normalized alignment coverage by the total number of mapped reads in the ChIP-Seq experiment. To assign scores to genes consisting of multiple isoforms, we computed a weighted average of the isoform scores, weighting isoforms by their FPKM.

To measure the relationship between the fRIP/input fold change and ChIP scores across all abundance levels, we first computed separate Lowess nonparametric regressions of FPKM versus ChIP score separately for genes bound and unbound in the fRIP-Seq experiment. Next, we integrated the difference between these two regression lines over the distribution of FPKM. This statistic is conceptually similar to computing the area of the region in between the two regression lines in the FPKM versus ChIP score plots, where we more heavily weight more likely FPKM levels.

### Ethics approval

Not applicable.

### Availability of data and materials

fRIP-Seq data are available through the Gene Expression Omnibus at accession GSE67963.

CLIP-Seq data were obtained for IMP1 from GSE21918, HNRNPH1 from GSE23694, HUR from GSE28865 and GSE29780, HNRNPU from GSE34491, and CTCF from GSE3554.

Depletion RNA-Seq data were obtained for CTCF from GSE44267, SUZ12 from GSE50177, HUR from GSE28865, and HNRNPU from ENCSR732ICL, and IMP1 from ENCSR629EWX.

## Additional files

Additional file 1: Supplementary Figures.
**Fig. S1** fRIP-Seq optimization. **Fig. S2** The effect of transcript localization on reassociation and fRIP-Seq enrichment. **Fig. S3** Validation of fRIP-Seq antibodies by western blot. **Fig. S4** ADAR preferentially binds to Alu elements and adjacent regions. **Fig. S5** fRIP-Seq broadly agrees with CLIP-Seq. **Fig. S6** fRIP-Seq targeted transcripts are affected by protein depletion. **Fig. S7** fRIP-Seq coverage displays light positional biases. **Fig. S8** Nuclear fRIP-Seq matches whole cell fRIP-Seq. **Fig. S9** The cohesin subunit STAG2 specifically binds a small cohort of transcripts encoding centrosome-localized proteins. **Fig. S10** lncRNA association with CAPs is more specific than CAP association with mRNAs. **Fig. S11** DNMT1 binds a GC-rich motif in lncRNAs. **Fig. S12** UUUUAAAA is a polarizing, conserved, 3’ motif. **Fig. S13** K-mers predict RNA binding preferences. **Fig. S14** Transposable elements correlate with RNA binding. **Fig. S15** Proteins with both fRIP and ChIP suggest a weak relationship. **Fig. S16** Chromatin marks correlate with gene abundance. **Fig. S17** fRIP versus chromatin mark correlations are FPKM-driven. **Fig. S18** Protein binding to RNA relates to local chromatin over the gene body. (PDF 22097 kb) Additional file 2: Table S1. Protein antibodies. (XLSX 46 kb) Additional file 3: Table S2 Gene clusters’ functional annotations. (XLSX 1364 kb)
