# Supplementary material for: Widespread RNA binding by chromatin-associated proteins
Source: Genome Biol. 2016 Feb 16;17:28. doi: 10.1186/s13059-016-0878-3 (PMC4756407; doi:10.1186/s13059-016-0878-3)
Supplement: Additional file 1: Supplementary Figures. — Fig. S1 fRIP-Seq optimization. Fig. S2 The effect of transcript localization on reassociation and fRIP-Seq enrichment. Fig. S3 Validation of fRIP-Seq antibodies by western blot. Fig. S4 ADAR preferentially binds to Alu elements and adjacent regions. Fig. S5 fRIP-Seq broadly agrees with CLIP-Seq. Fig. S6 fRIP-Seq targeted transcripts are affected by protein depletion. Fig. S7 fRIP-Seq coverage displays light positional biases. Fig. S8 Nuclear fRIP-Seq matches whole cell fRIP-Seq. Fig. S9 The cohesin subunit STAG2 specifically binds a small cohort of transcripts encoding centrosome-localized proteins. Fig. S10 lncRNA association with CAPs is more specific than CAP association with mRNAs. Fig. S11 DNMT1 binds a GC-rich motif in lncRNAs. Fig. S12 UUUUAAAA is a polarizing, conserved, 3’ motif. Fig. S13 K-mers predict RNA binding preferences. Fig. S14 Transposable elements correlate with RNA binding. Fig. S15 Proteins with both fRIP and ChIP suggest a weak relationship. Fig. S16 Chromatin marks correlate with gene abundance. Fig. S17 fRIP versus chromatin mark correlations are FPKM-driven. Fig. S18 Protein binding to RNA relates to local chromatin over the gene body. (PDF 22097 kb) [file 13059_2016_878_MOESM1_ESM.pdf]

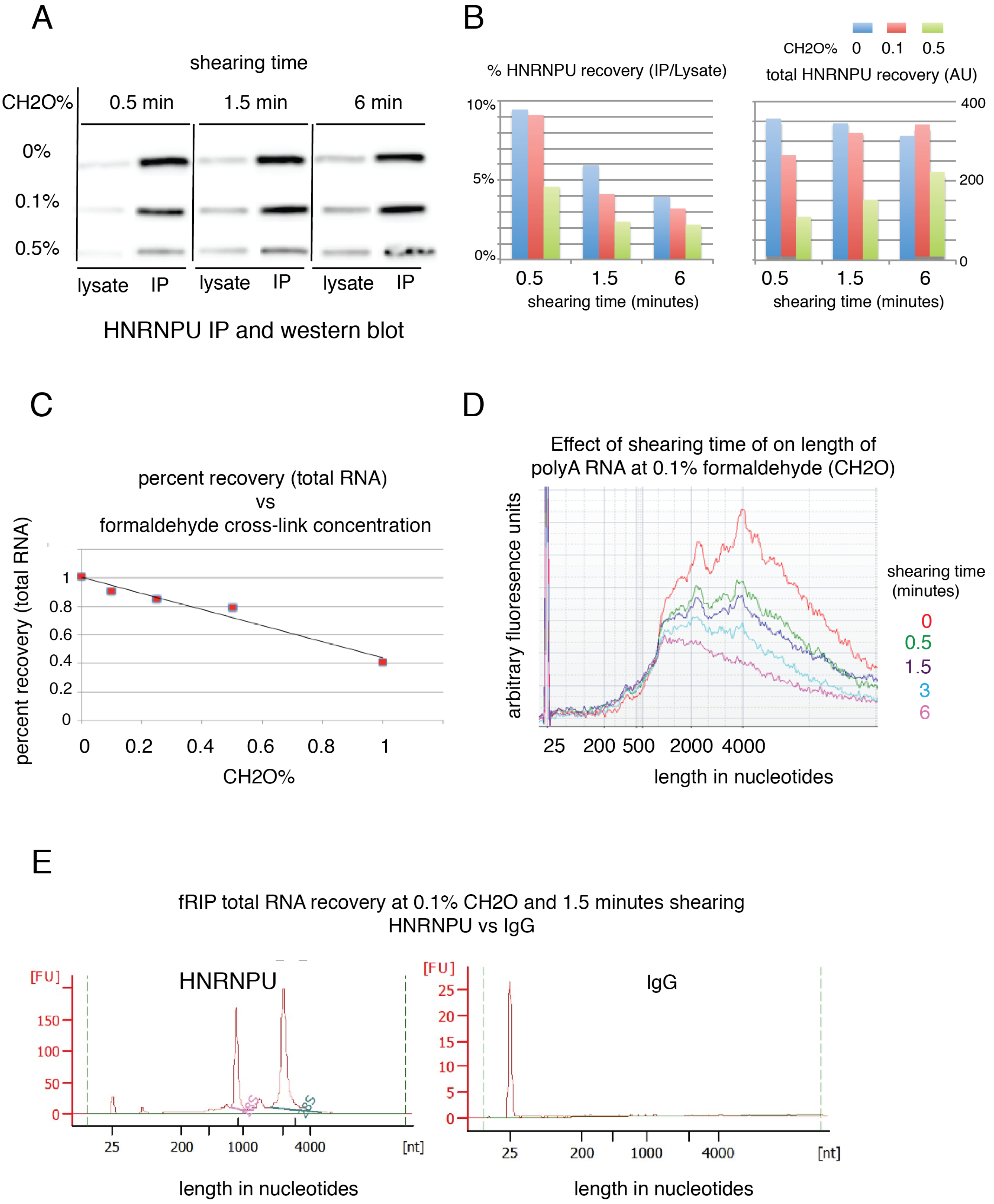
A
shearing time
CH2O%
0.5 min
1.5 min
6 min
0%
0.1%
0.5%
lysate
IP
lysate
IP
lysate
IP
HNRNPU IP and western blot
B
CH2O%
0
0.1
0.5
% HNRNPU recovery (IP/Lysate)
10%
5%
0%
0.5
1.5
6
shearing time (minutes)
total HNRNPU recovery (AU)
400
200
0
0.5
1.5
6
shearing time (minutes)
C
percent recovery (total RNA)
vs
formaldehyde cross-link concentration
percent recovery (total RNA)
1
0.8
0.6
0.4
0.2
0
0
0.2
0.4
0.6
0.8
1
CH2O%
D
Effect of shearing time of on length of
polyA RNA at 0.1% formaldehyde (CH2O)
arbitrary fluoresence units
25
200
500
2000
4000
length in nucleotides
shearing time
(minutes)
0
0.5
1.5
3
6
E
fRIP total RNA recovery at 0.1% CH2O and 1.5 minutes shearing
HNRNPU vs IgG
[FU]
HNRNPU
150
100
50
0
25
200
1000
4000
[nt]
length in nucleotides
[FU]
IgG
25
20
15
10
5
0
25
200
1000
4000
[nt]
length in nucleotides

Figure S1: fRIP-Seq optimization

We tested the efficiency and integrity of HNRNPU and RNA recovery respectively over a range of the two immunoprecipitation conditions, formaldehyde concentration (cross-linking), and sonication (shearing). (A) Higher amounts of HNRNPU were recovered in native (0%) and lower formaldehyde concentrations (0.1%) in both lysate and immunoprecipitation reactions than with 0.5% formaldehyde. (B) Longer sonication times mitigated higher formaldehyde concentrations and increased yield, as quantified using densitometry to calculate relative and total yield. (C) Total RNA recovery was less efficient with increasing formaldehyde concentration. (D) Longer sonication resulted in smaller sized polyA selected RNA at 0.1% formaldehyde. (E) Total RNA recovery associated with HNRNPU compared to total RNA recovery associated with the IgG negative control.

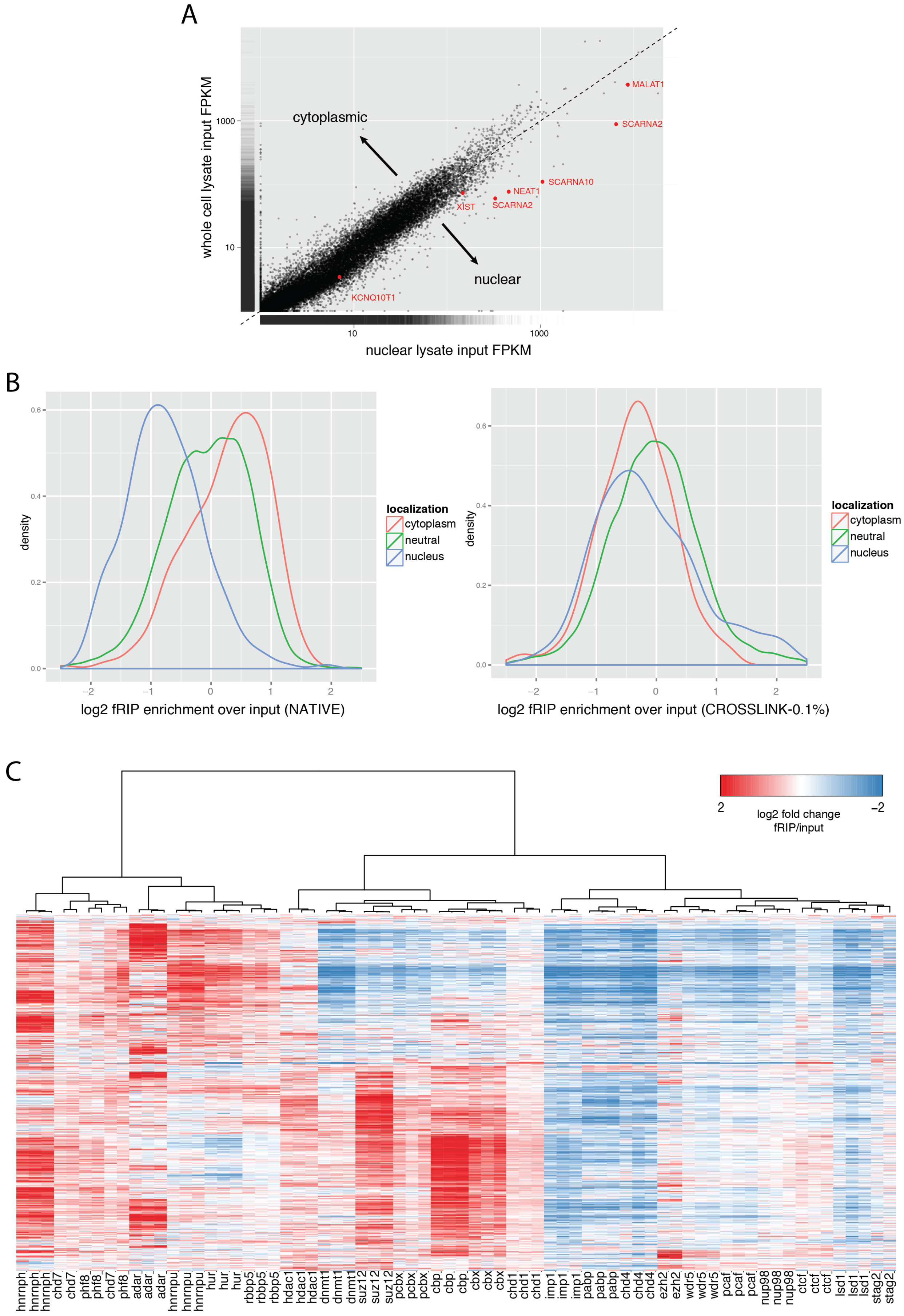
A
whole cell lysate input FPKM
nuclear lysate input FPKM
cytoplasmic
nuclear
MALAT1
SCARNA2
SCARNA10
NEAT1
XIST
SCARNA2
KCNQ1OT1
B
density
log2 fRIP enrichment over input (NATIVE)
log2 fRIP enrichment over input (CROSSLINK-0.1%)
localization
cytoplasm
neutral
nucleus
C
log2 fold change
fRIP/input
hnrnph hnrnph hnrnph chd7 chd7 phf8 phf8 chd7 phf8 adar adar adar hnrnpu hnrnpu hnrnpu hur hur hur rbbp5 rbbp5 rbbp5 hdac1 hdac1 hdac1 dnmt1 dnmt1 dnmt1 suz12 suz12 suz12 pcbx pcbx pcbx cbp cbp cbp cbx cbx cbx chd1 chd1 chd1 imp1 imp1 imp1 pabp pabp pabp chd4 chd4 chd4 ezh2 ezh2 wdr5 wdr5 wdr5 pcaf pcaf pcaf nup98 nup98 nup98 ctcf ctcf ctcf lsd1 lsd1 lsd1 stag2 stag2

Figure S2: The effect of transcript localization on reassociation and fRIP-Seq enrichment

(A) Scatter plot of abundance (FPKM) generated by RNA-Seq from nuclear lysate (x-axis) versus whole cell lysate (y-axis) in K562 cells. Several canonical nuclear-localized transcripts are highlighted in red. (B) Density (y-axis) plots of HNRNPU log2 fold enrichment over input for nuclear (blue), neutral (green), and cytoplasmic (red) transcripts under native RIP (left) versus fRIP 0.1% formaldehyde crosslinking conditions (right). Under native conditions we observed higher enrichment of cytoplasmically-localized transcripts than under cross-linked conditions, suggesting that the fRIP conditions reduce re-association and post-lysis mixing. (C) Heat map and hierarchical clustering of log2 fold fRIP enrichment over whole cell input for the 1300 genes enriched in the nucleus with a p-value of < 0.01. Depletion of these transcripts from fRIP-Seq for cytosolic proteins is evident for IMP1 and PABP. Likewise, fRIP-Seq of the predominantly nuclear HNRNPH reveals enrichment for these transcripts as a whole. However, most proteins and CAPs we surveyed with fRIP-Seq do not enrich or deplete for transcripts as a function of their localization, suggesting that the nuclear localization of CAPs is not the primary contributing factor to the transcripts we observed as being enriched or depleted.

Antibody: ADAR
Vendor: Sigma
Part number: HPA003890
Lot number: HPA003890
Expected size: ~130 kD

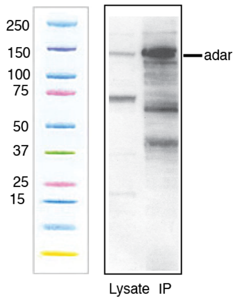

Antibody: CBP
Vendor: Cell Signaling
Part number: 7389S
Lot number: 1
Expected size: ~300 kD

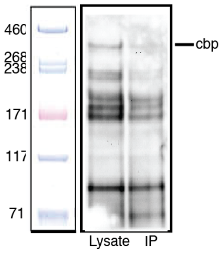

Antibody: CHD1
Vendor: Cell Signaling
Part number: 4351S
Lot number: 1
Expected size: 220

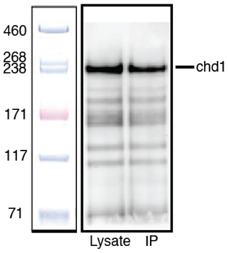

Antibody: CHD4
Vendor: Abcam
Part number: ab70469
Lot number: GR104037-5
Expected size: ~260 kD

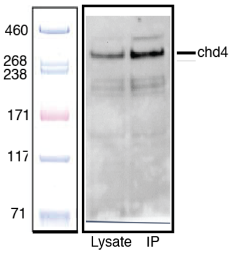

Antibody: CHD7
Vendor: Bethyl
Part number: A301-223A
Lot number: A301-223A-1
Expected size:
multiple large isoforms:

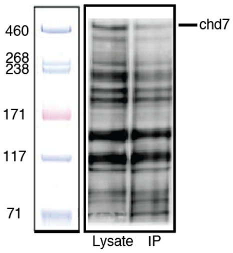

Antibody: DNMT1
Vendor: Bethyl
Part number: ab13537
Lot number: GR118996-2
Expected size: ~180 kD

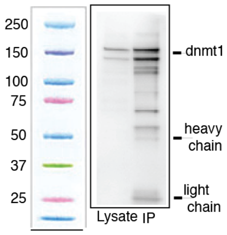

Antibody: HDAC1
Vendor: Cell Signaling
Part number: 5356S
Lot number: 3
Expected size: ~62 kD

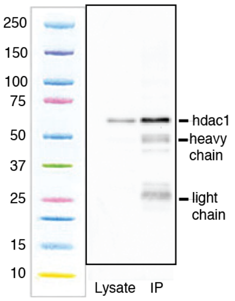

Antibody: HNRNPH1
Vendor: Bethyl
Part number: A300-511A
Lot number: A300-511A-2
Expected size: ~60 kD

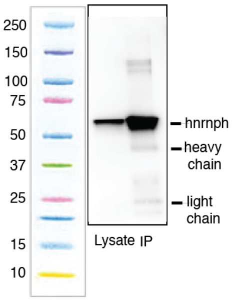

Antibody: HNRNPU
Vendor: Abcam
Part number: ab20666
Lot number: GR119286-1
Expected size: ~117 kD

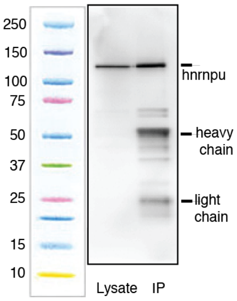

Antibody: HUR
Vendor: Santa Cruz
Part number: sc-5483
Lot number: 5
Expected size: ~35 kD

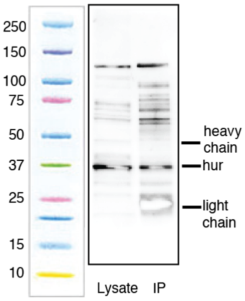

Antibody: IGF2BP1/IMP1
Vendor: Cell Signaling
Part number: 8482S
Lot number: 1
Expected size: ~64 kD

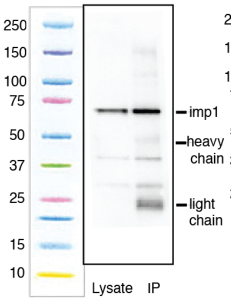

Antibody: KDM1/LSD1
Vendor: Abcam
Part number: ab17721
Lot number: GR137609-1
Expected size: ~100 kD

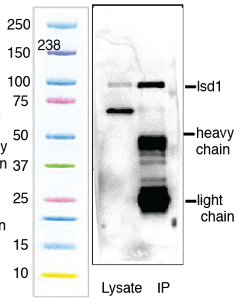

Antibody: NUP98
Vendor: Cell Signaling
Part number: 2598S
Lot number: 2
Expected size: ~98 kD

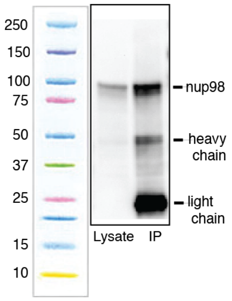

IP-1 Antibody: PCAF
Vendor: Bethyl
Part number: A300-666A
Lot number: 1

IP-2 Antibody: PCAF
Vendor: Cell Signaling
Part number: 3378S
Lot number: 1

Expected size: ~93 kD

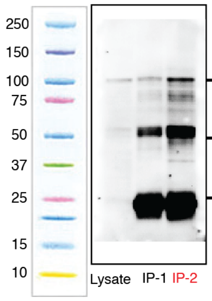

Antibody: phospho-Hp1y/CBX3(ser83)
Vendor: Cell Signaling
Part number: 2600S
Lot number: 2
Expected size: ~22 kD

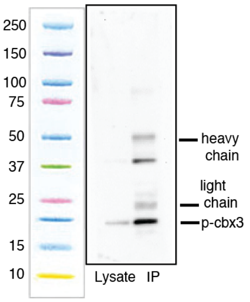

Antibody: PHF8
Vendor: Bethyl
Part number: A301-772A
Lot number: A301-772A-1
Expected size: ~130-150 kD

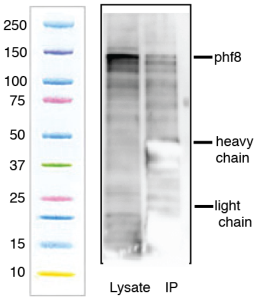

IP-1 Antibody: RBBP5
Vendor: Bethyl
Part number: A300-109A
Lot number: 2

IP-2 Antibody: RBBP5
Vendor: Cell Signaling
Part number: 13171S
Lot number: 1

Expected size: ~70 kD

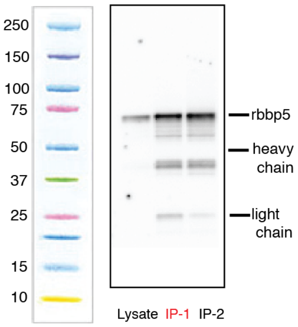

Antibody: SETD2
Vendor: Sigma-Aldrich
Part number: HPA042451-100UL
Lot number: B91845
Expected size:~290 kD

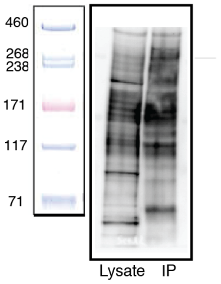

Antibody: STAG2
Vendor: Cell Signaling
Part number: 5882S
Lot number: 1
Expected size: ~141 kD

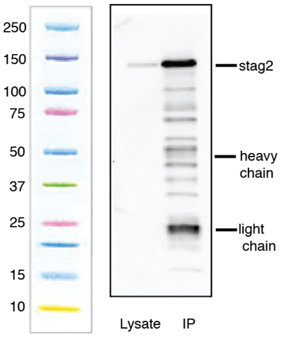

Antibody: SUZ12
Vendor: Cell Signaling
Part number: 3737S
Lot number: 3
Expected size: ~83 kD

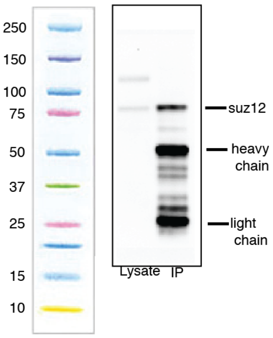

IP-1 Antibody: WDR5
Vendor: Bethyl
Part number: A302-429A
Lot number: A302-429A-1

IP-2 Antibody: WDR5
Vendor: Bethyl
Part number: A302-430A
Lot number: A302-430A-1

IP-3 Antibody: WDR5
Vendor: Cell Signaling
Part number: 13105
Lot number: 1

Expected size: ~37 kD

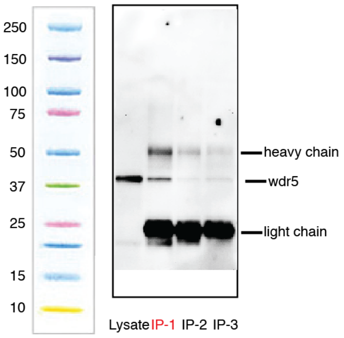

Figure S3: Validation of fRIP-Seq antibodies by western blot.

For antibodies not previously validated by ENCODE, we performed “mini” fRIP experiments using 1/10 volume of lysate and antibody. We separated using SDS-PAGE and western blotted for the target fRIP protein. We ran each mini fRIP alongside 10 micrograms of whole cell lysate. In each panel, we noted the expected size alongside the specific band(s), as well as bands running at the expected size for IgG heavy and light chains. Some of the antibodies for larger molecular weight proteins recognize multiple large isoforms and/or smaller degradation product bands. For several proteins, we tested multiple antibodies and note the final antibody used in red text. SETD2 is an example of a rejected antibody that did not recognize a distinct band at the expected size and recognized thousands of other bands.

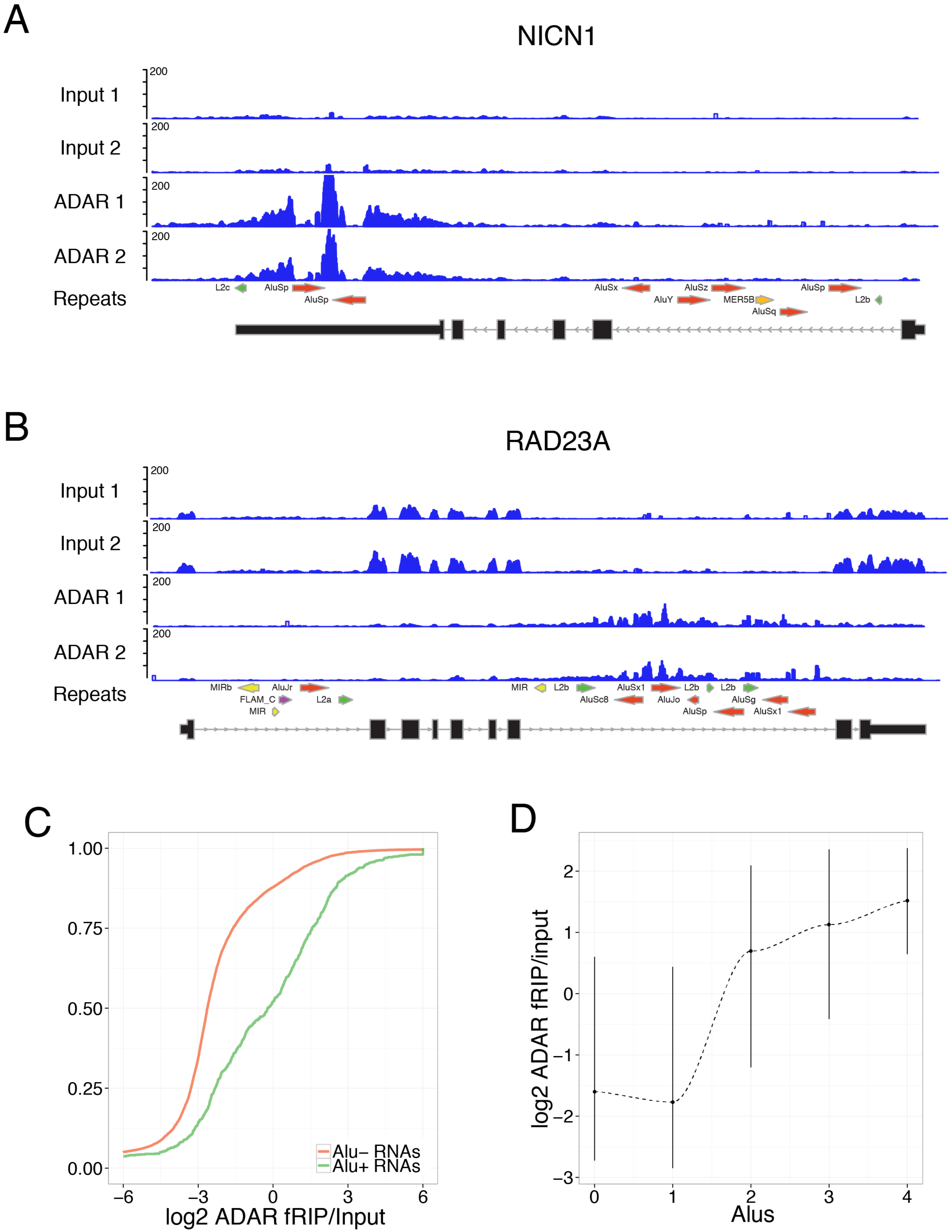
A
NICN1
Input 1
Input 2
ADAR 1
ADAR 2
Repeats
200
L2c
AluSp
AluSp
AluSx
AluY
AluSz
MER5B
AluSq
AluSp
L2b
B
RAD23A
Input 1
Input 2
ADAR 1
ADAR 2
Repeats
200
MIRb
AluJr
FLAM_C
MIR
L2a
MIR
L2b
AluSx1
L2b
L2b
AluSc8
AluJo
AluSg
AluSp
AluSx1
C
1.00
0.75
0.50
0.25
0.00
−6
−3
0
3
6
log2 ADAR fRIP/Input
Alu− RNAs
Alu+ RNAs
D
log2 ADAR fRIP/input
2
1
0
−1
−2
−3
0
1
2
3
4
Alus

Figure S4: ADAR preferentially binds to Alu elements and adjacent regions.

We plotted examples of ADAR association with Alu repeats captured with fRIP-Seq. For each gene, we display input RNA-Seq coverage above ADAR fRIP-Seq coverage. Annotation tracks for RepeatMasker SINE elements (Alu instances are in red) and GENCODE transcript models are below the coverage tracks. ADAR binds Alu elements in (A) NICN1 and (B) RAD23A. (C) Genome-wide, genes containing Alu elements have greater fRIP/input fold changes than genes without them, shown here as the empirical cumulative distribution functions of each class. (D) Genes containing more Alu elements have greater fold changes, shown as the 25th, 50th, and 75th percentile of the fRIP/input fold change distribution for each Alu count.

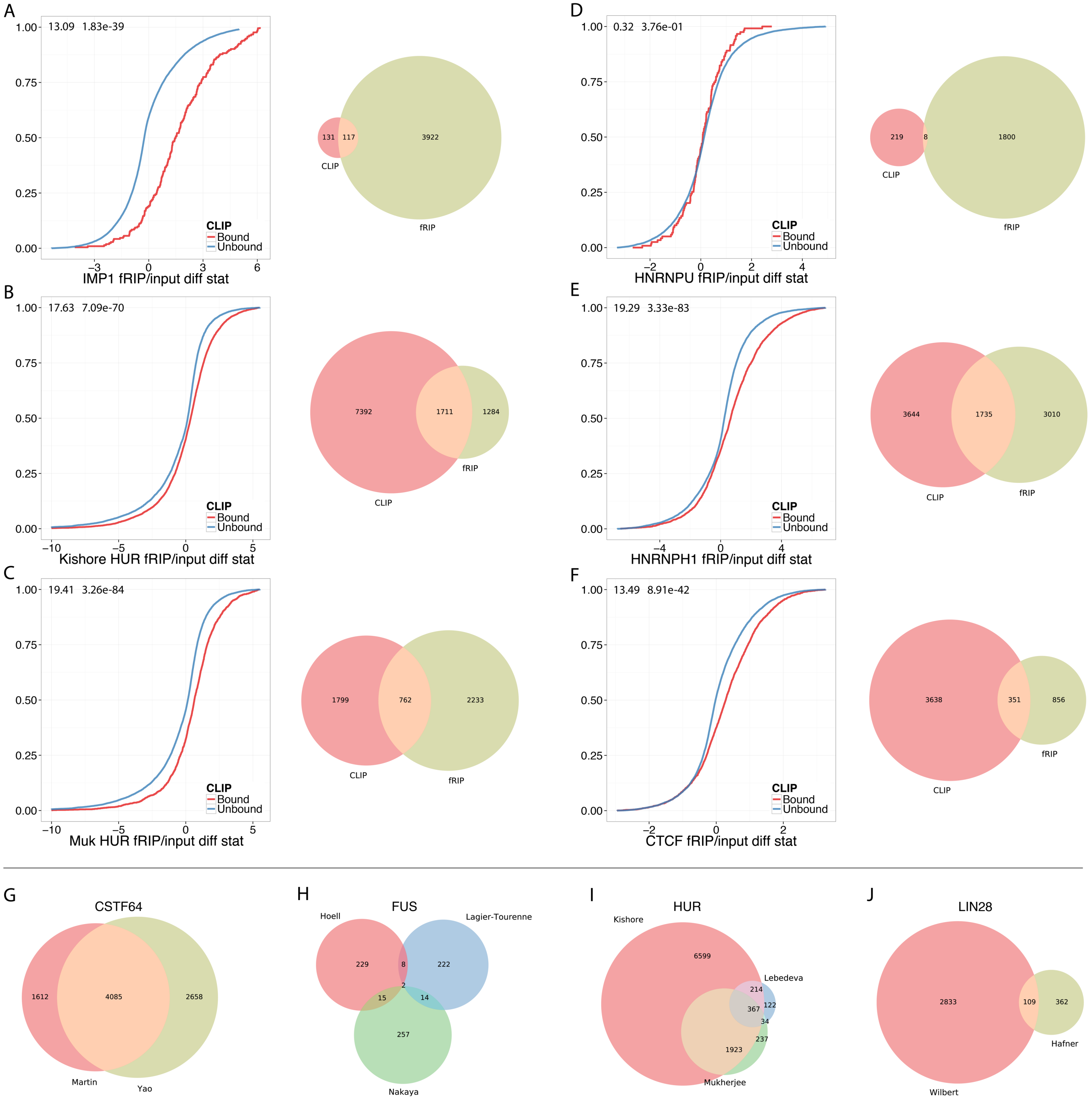

Figure S5: fRIP-Seq broadly agrees with CLIP-Seq.

We compared Cuffdiff differential expression test statistics for CLIP-Seq bound genes to all unbound genes. For CTCF, HNRNPH1, IMP1 and two versions of HUR, CLIP-Seq bound RNAs had significantly greater difference statistics than unbound RNAs (p-values < 1e-37), shown as the empirical cumulative distribution functions of the statistic for each set. This enrichment was apparent despite all CLIP-Seq experiments having been performed in different cell types (HEK293 for IMP1, HUR, and HNRNPH1; HeLa for HNRNPU; U2OS for CTCF).

Mann-Whitney U test Z-scores and p-values are specified in the upper left corners. The difference was insignificant for HNRNPU, for which only 227 targeted genes could be discerned from the CLIP-Seq data, of which only 138 were expressed in our K562 cells. Venn diagrams compare and contrast the CLIP-Seq bound RNAs with significantly bound RNAs identified by fRIP-Seq. The bottom row depicts analogous Venn diagram comparisons for CLIP-Seq performed on the same protein in different projects (labeled by manuscript first author), demonstrating that the fRIP/CLIP overlaps are within the bounds of CLIP/CLIP overlaps.

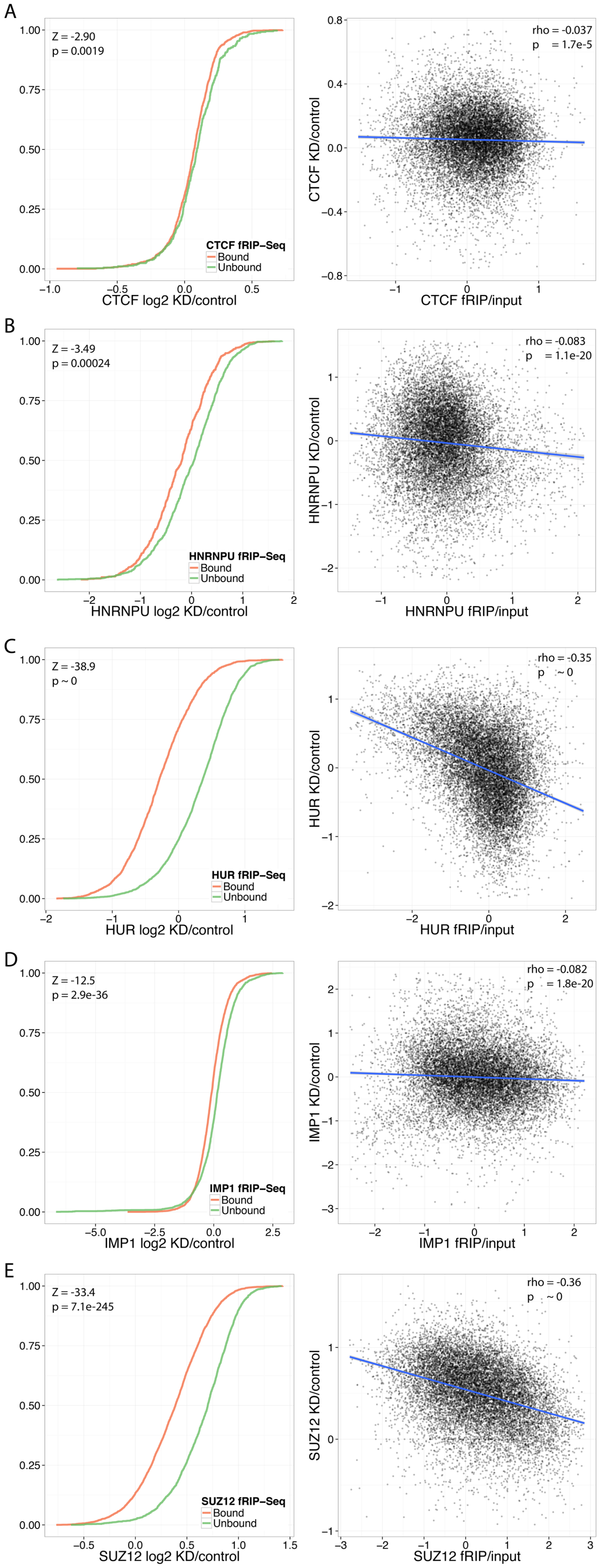

Figure S6: fRIP-Seq targeted transcripts are affected by protein depletion.
We downloaded and analyzed publicly available RNA-Seq measuring gene expression after depletion of five of our proteins. In the left column of plots, we classified all genes as significantly bound or unbound by fRIP-Seq and plotted the empirical cumulative distribution function of log2 knockdown/control fold changes for each set. Mann-Whitney U tests confirmed that the abundance of bound genes was significantly altered for each protein. In the right column, we plotted fold changes between the two experiments for all genes. Spearman correlations indicate a significant relationship between the two in every case.

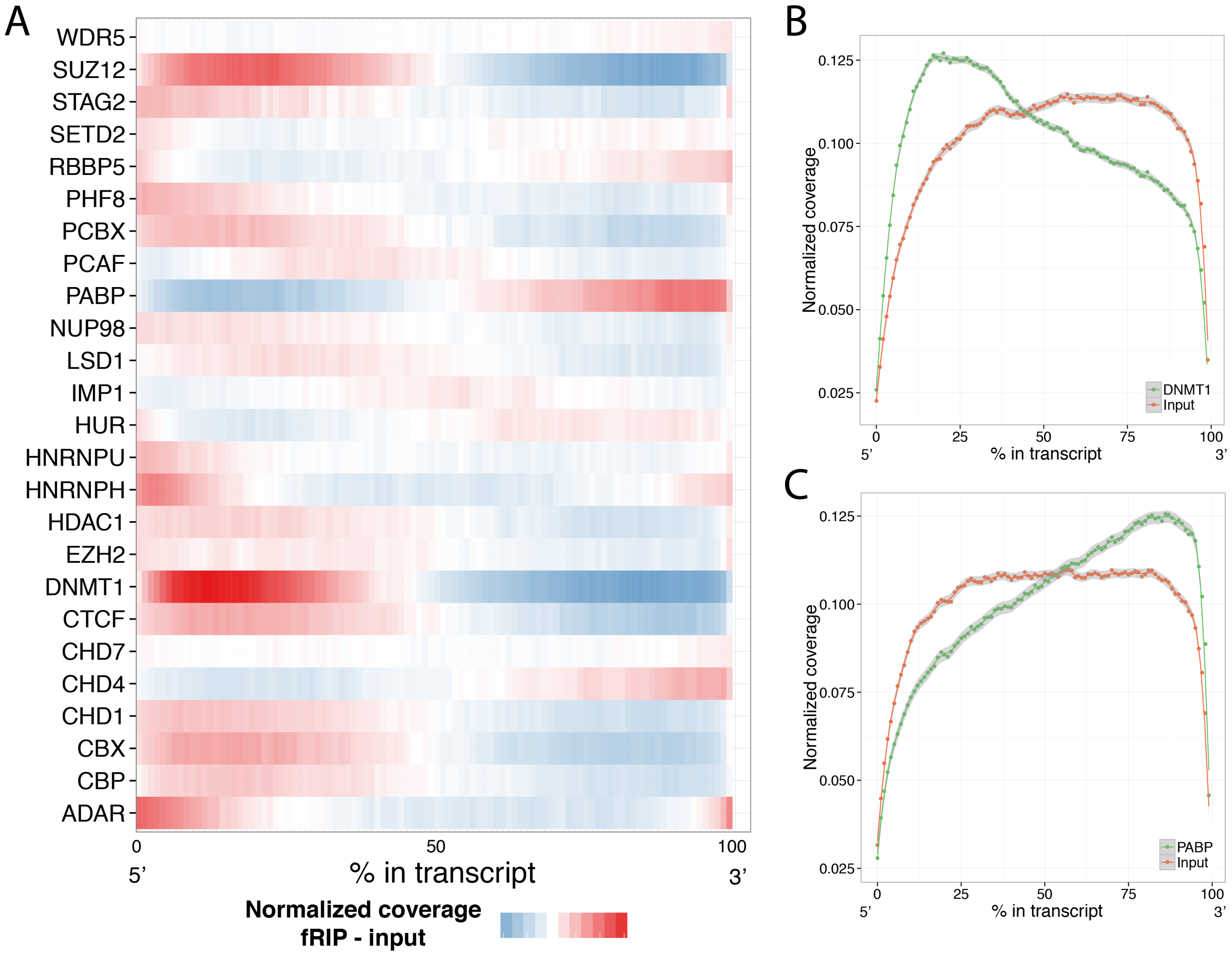

Figure S7: fRIP-Seq coverage displays light positional biases.
Though fRIP-Seq does not provide binding site resolution, read coverage for some experiments trended towards one end of the transcript. We computed alignment coverage in 100 uniformly-sized bins for each gene for both the fRIP-Seq and input sequencing for every protein. After normalizing bin coverages to sum to one, we plotted the values in (B,C) and the difference between fRIP and input as a heat map (A). Reflecting known biology, PABP binds 3' transcript ends. DNMT1 and SUZ12 tended towards 5' ends.

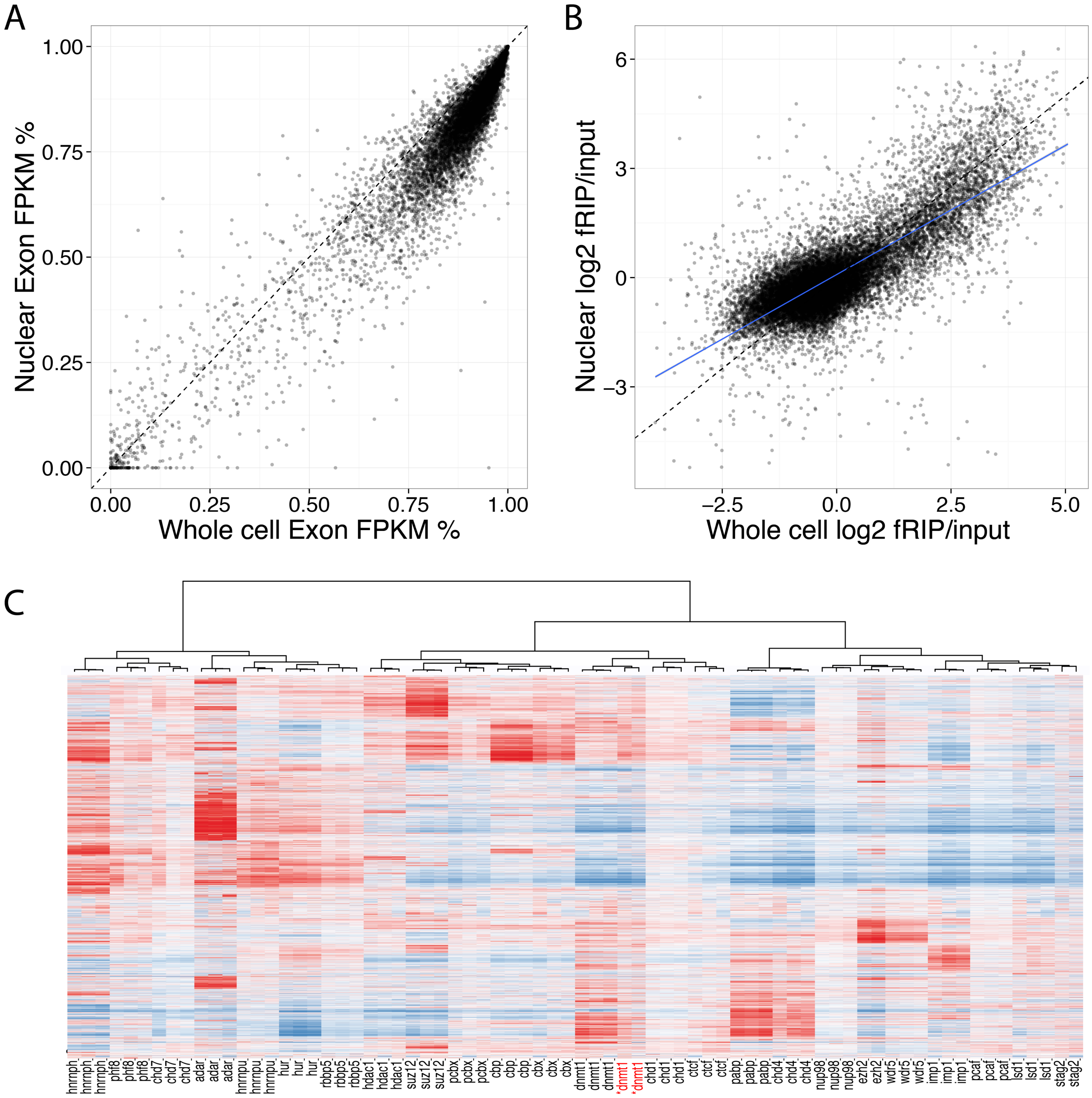

Figure S8: Nuclear fRIP-Seq matches whole cell fRIP-Seq.
(A) We computed the proportion of a genes' FPKM assigned to exon isoforms versus unspliced pre-RNA isoforms (Methods) in whole cell input and nuclear fraction input. As expected, transcripts in the nuclear fraction tended to be slightly less spliced. However, the nuclear fraction still contained primarily spliced transcripts, suggesting that fRIP-mapped interactions with mature spliced transcripts regularly occur in the nucleus. (B) fRIP-Seq log2 fold changes for DNMT1 in a nuclear fraction versus whole cell match well, suggesting that whole cell interactions are primarily nuclear. (C) Further

supporting their concordance, whole cell (black font) and nuclear (red font with asterisk) DNMT1 fRIP-Seq replicates cluster together relative to all other fRIPs performed in a hierarchical clustering of log2 fold changes for the ~25,000 genes present in at least one condition.

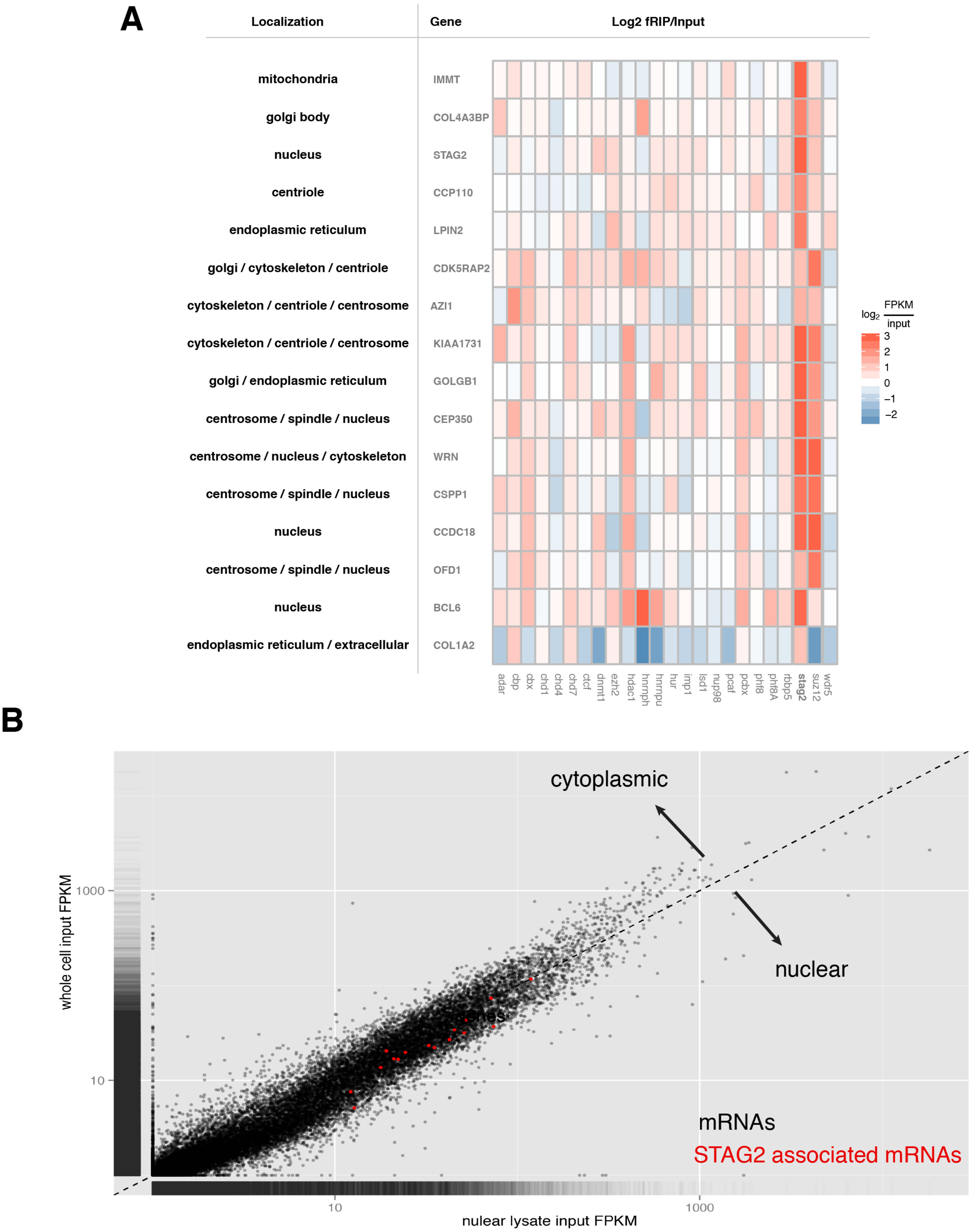

A
Localization
Gene
Log2 fRIP/Input
mitochondria
golgi body
nucleus
centriole
endoplasmic reticulum
golgi / cytoskeleton / centriole
cytoskeleton / centriole / centrosome
cytoskeleton / centriole / centrosome
golgi / endoplasmic reticulum
centrosome / spindle / nucleus
centrosome / nucleus / cytoskeleton
centrosome / spindle / nucleus
nucleus
centrosome / spindle / nucleus
nucleus
endoplasmic reticulum / extracellular
IMMT
COL4A3BP
STAG2
CCP110
LPIN2
CDK5RAP2
AZI1
KIAA1731
GOLGB1
CEP350
WRN
CSPP1
CCDC18
OFD1
BCL6
COL1A2
adar
cbp
cbx
chd1
chd4
chd7
ctcf
dnmt1
ezh2
hdac1
hnrnph
hnrnpu
hur
imp1
lsd1
nup98
pcaf
pcbx
phf8
phf8A
rbbp5
stag2
suz12
wdr5
log2 FPKM / input
3
2
1
0
−1
−2
B
cytoplasmic
nuclear
mRNAs
STAG2 associated mRNAs
whole cell input FPKM
1000
10
nulear lysate input FPKM
10
1000

Figure S9: The cohesin subunit STAG2 specifically binds a small cohort of transcripts encoding centrosome-localized proteins.

(A) Heat map of log2 fRIP/input for transcripts significantly enriched in STAG2 fRIP-Seq experiments. The variable fold changes of the other proteins for these genes precludes the possibility of a protocol bias towards the set. Subcellcular localizations obtained from the COMPARTMENTS database (Binder et al. 2014) indicate that STAG2-bound transcripts encode for proteins that localize to centrosomes, centrioles, spindles, and membrane bound organelles, in line with known functional roles of STAG2. (B) STAG2 enrichment does not appear to be driven by STAG2 protein nuclear localization. In a scatter plot of gene abundance (FPKM) generated by RNA-Seq from nuclear lysate (x-axis) versus whole cell lysate (y-axis) in K562 cells, STAG2-enriched transcripts (in red) lack a bias towards one or the other.

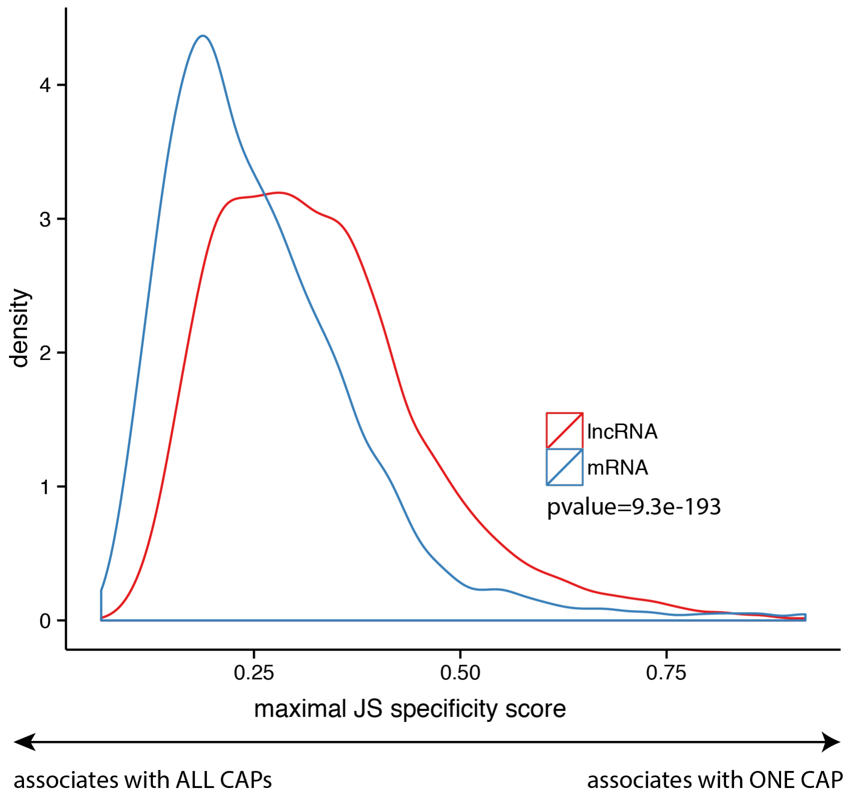

Figure S10: lncRNA association with CAPs is more specific than CAP association with mRNAs.

Both mRNAs and lncRNAs associate with CAPs, but lncRNAs associate with fewer CAPs on average. We plotted the distributions of maximal CAP specificity scores calculated for an abundance-matched set of mRNAs (blue) and lncRNAs (red). The specificity distribution for lncRNAs was higher than for mRNAs using the Wilcoxon rank sum test (p-value < 9.3e-193).

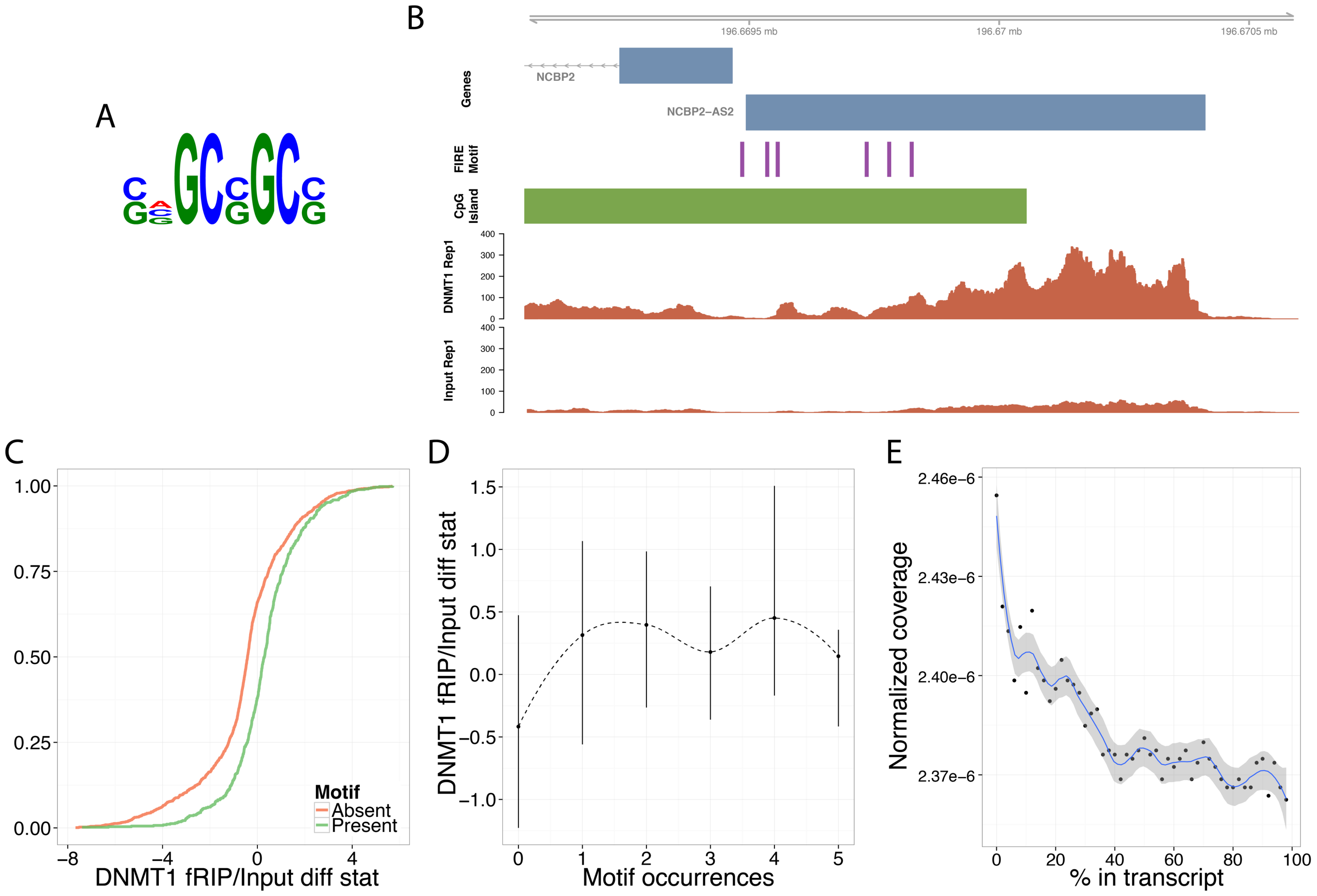

Figure S11: DNMT1 binds a GC-rich motif in lncRNAs.

(A) We discovered an 8 nt GC-rich motif that had high mutual information with the DNMT1 fRIP/input differential expression statistic for lncRNAs. (B) For example, the motif occurs multiple times in a lncRNA antisense of the mRNA NCBP2, overlapping the CpG island of its promoter. (C) Empirical cumulative distribution functions of the DNMT1 fRIP/input differential expression statistic, separated by lncRNAs with and without the motif, demonstrate its strong effect. (D) The 25th, 50th, and 75th quantiles of the statistic distribution for transcripts with the specified numbers of motifs indicate that presence/absence is sufficient to explain the motif's effect. (E) As exemplified by the motif instances in NCBP2-AS2, coverage of the motif is enriched at the 5' ends of transcripts. We normalized motif coverage counts by the number of transcripts.

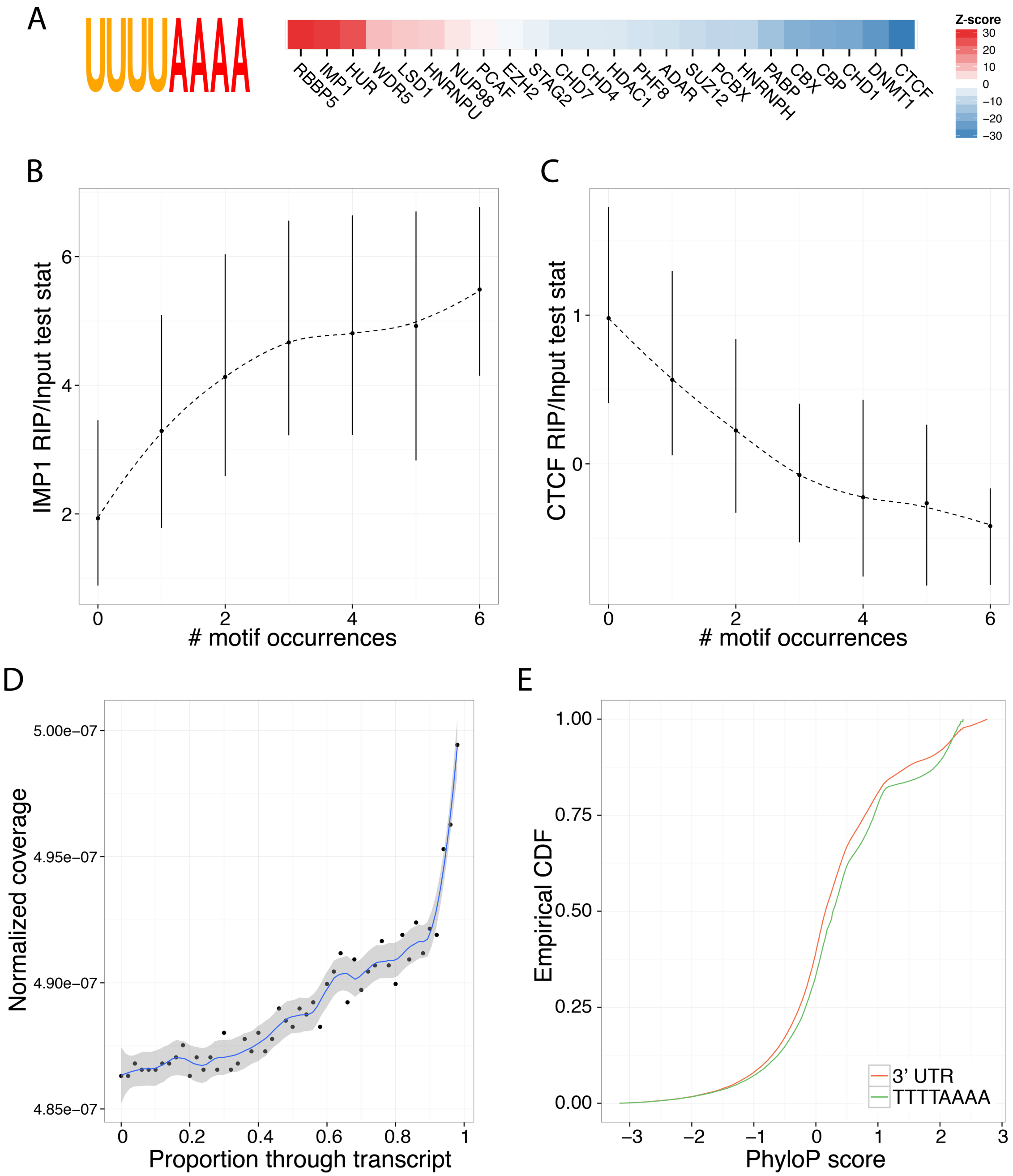

Figure S12: UUUUAAAA is a polarizing, conserved, 3' motif.

(A) The RBPs responded strongly to the motif UUUUAAAA and slight variations of it, shown as the Mann-Whitney U test Z-score comparing Cuffdiff differential expression statistics for genes with and without the motif. Among the strongest effects were (B) IMP1's preference for and (C) CTCF's avoidance of transcripts containing the motif, shown here as the 25th, 50th, and 75th percentile of the fRIP/input statistic distributions for transcripts with 0-6 motif occurrences. (D) The motif tended to occur towards the 3' ends of transcripts, shown here as the average coverage over transcripts, each divided individually into 50 equally sized bins. (E) The distribution of PhyloP scores for motif occurrences in 3' UTRs was shifted towards greater scores relative to all 3' UTR nucleotides.

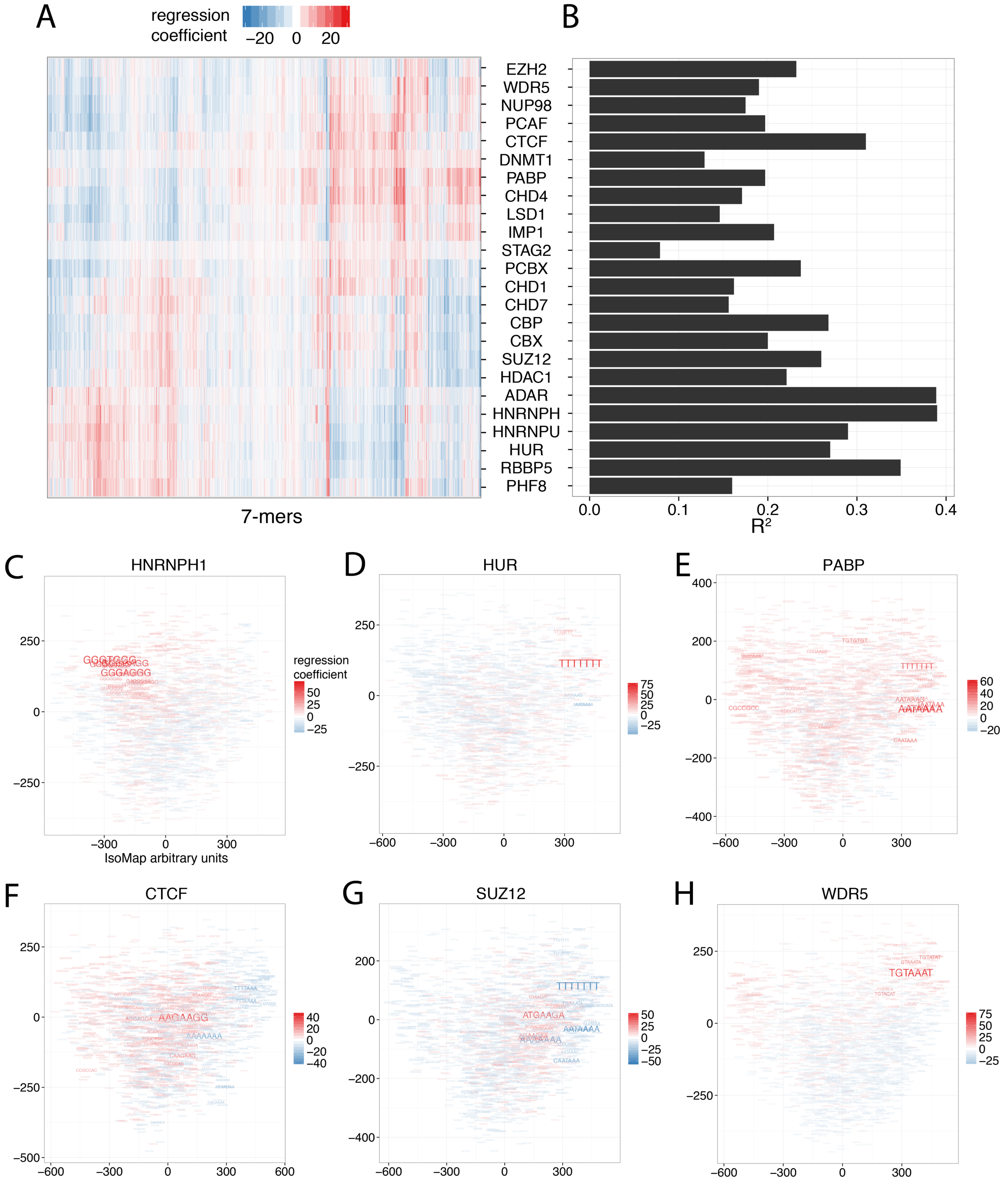

A
regression coefficient
−20 0 20
EZH2
WDR5
NUP98
PCAF
CTCF
DNMT1
PABP
CHD4
LSD1
IMP1
STAG2
PCBX
CHD1
CHD7
CBP
CBX
SUZ12
HDAC1
ADAR
HNRNPH
HNRNPU
HUR
RBBP5
PHF8
7-mers
B
0.0 0.1 0.2 0.3 0.4
R²
C
HNRNPH1
GGGAGGG
IsoMap arbitrary units
D
HUR
TTTTTTT
E
PABP
AATAAAA
F
CTCF
AAGAAGG
AAAAAAA
G
SUZ12
TTTTTTT
ATGAAGA
AATAAAA
H
WDR5
TGTAAAT

Figure S13: K-mers predict RNA binding preferences.

We fit a linear regression model, in which every 7-mer was assigned a coefficient that was optimized to best predict a transcript's fRIP/input fold change from its 7-mer counts vector. (A) Clustering all proteins by these coefficient vectors displayed the relationships between their binding preferences. (B) On the right, the R^2 values (proportion of variance explained by the model) indicate the extent to which a 7-mer based model can predict binding for each protein. The greatest accuracies were achieved for ADAR, which focused on Alu 7-mers, and HNRNPH1, which focused on G-rich 7-mers. To better visualize the most important 7-mers weighted by the model, we plotted the coefficients on a 2D surface with related 7-mers nearby. The placement of 7-mers on the 2D surface was computed by a nonparametric dimension reduction approach called IsoMap on the co-occurrence vectors of the 7-mers. In doing so, we can see the importance of (C) G-rich 7-mers to the HNRNPH1 model, (D) U-rich 7-mers to the HUR model, and (E) 7-mers containing the polyadenylation signal AAUAAA for PABP. Among CAPs with less studied sequencing composition preferences, we see AG-rich 7-mers, but not AU-rich 7-mers for (F) CTCF and (G) SUZ12, (H) UGUAAAU and related 7-mers for WDR5.

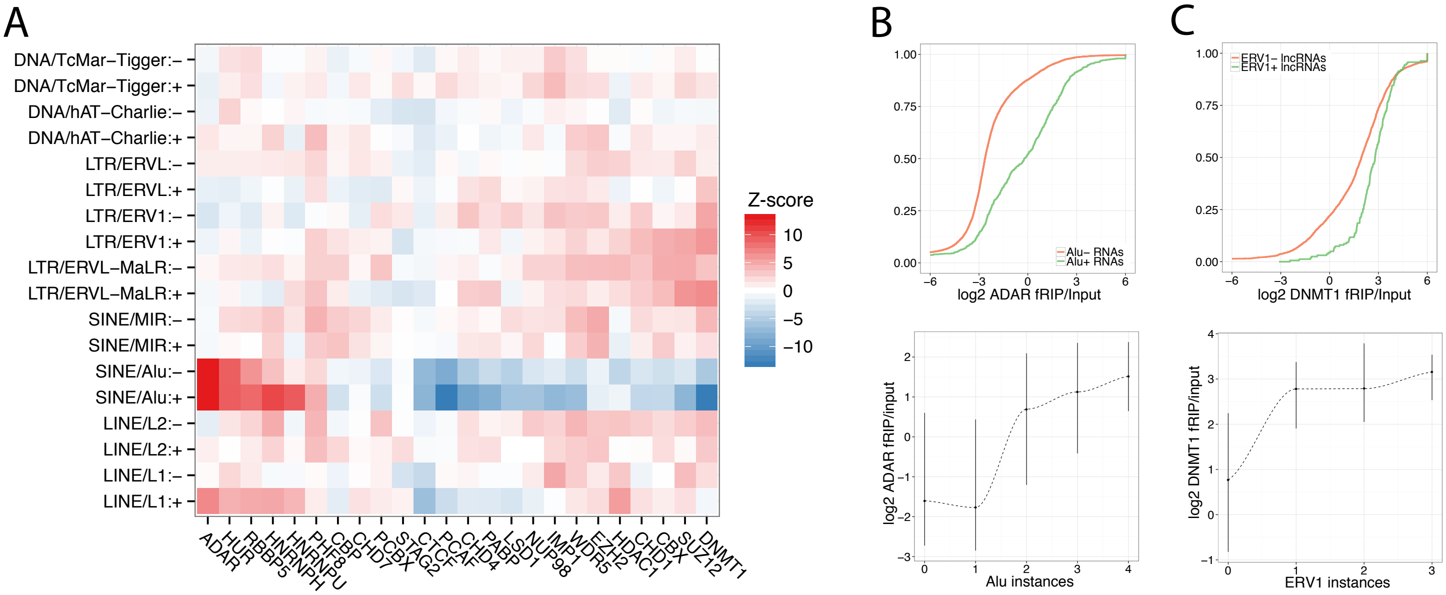

Figure S14: Transposable elements correlate with RNA binding.

(A) Mann-Whitney U test Z-scores comparing fRIP/input fold changes for transcripts with and without specific transposable element (TE) families revealed many significant associations. Only the most expressed transcript of every gene was considered. (B) Empirical cumulative distribution functions of RNAs with and without Alu elements demonstrate the strong role of Alu in determining ADAR binding. Below, the 25th, 50th, and 75th percentiles of the fRIP/input distribution indicate that binding increases with two or more Alu's, reflecting a known preference for ADAR to bind double-stranded Alu sequences folded on themselves. (C) A DNMT1 preference for sense-strand ERVs was apparent for all RNAs, but primarily limited to lncRNAs.

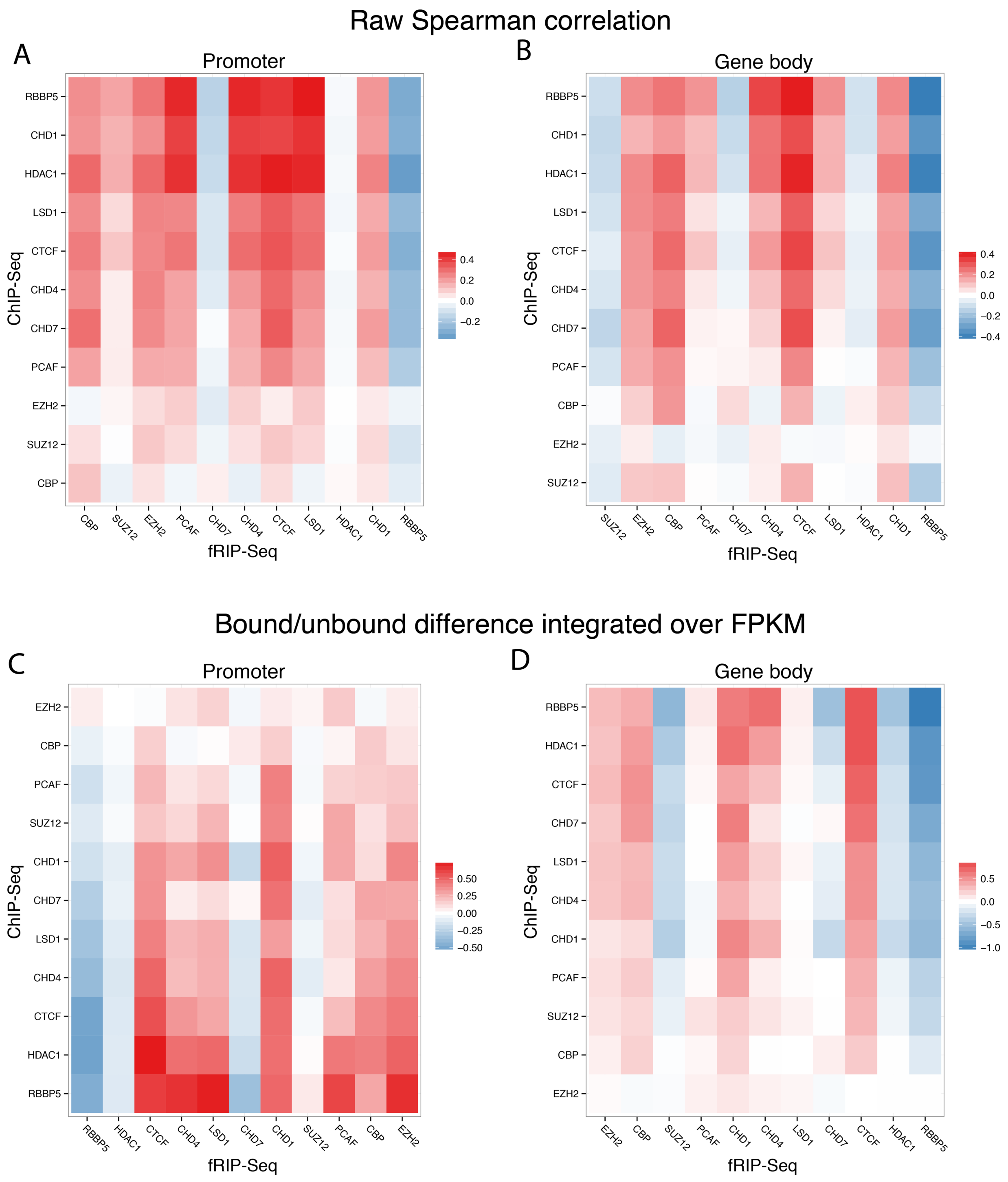

Figure S15: Proteins with both fRIP and ChIP suggest a weak relationship.
Over all genes, we computed the Spearman correlation between fRIP-Seq/input and ChIP/input for all shared proteins in the (A) promoter and (B) gene body. Because both fRIP-Seq/input and ChIP-Seq/input correlate with FPKM, these correlations are FPKM-driven and challenging to interpret. Thus, we also plotted a statistic measuring the difference in ChIP-Seq/input across FPKM levels between bound and unbound RNAs (Methods) for the (C) promoter and (D) gene body. In both views, a relationship between fRIP-Seq and ChIP-Seq is not apparent, suggesting that factors other than DNA occupancy are more important to RNA binding.

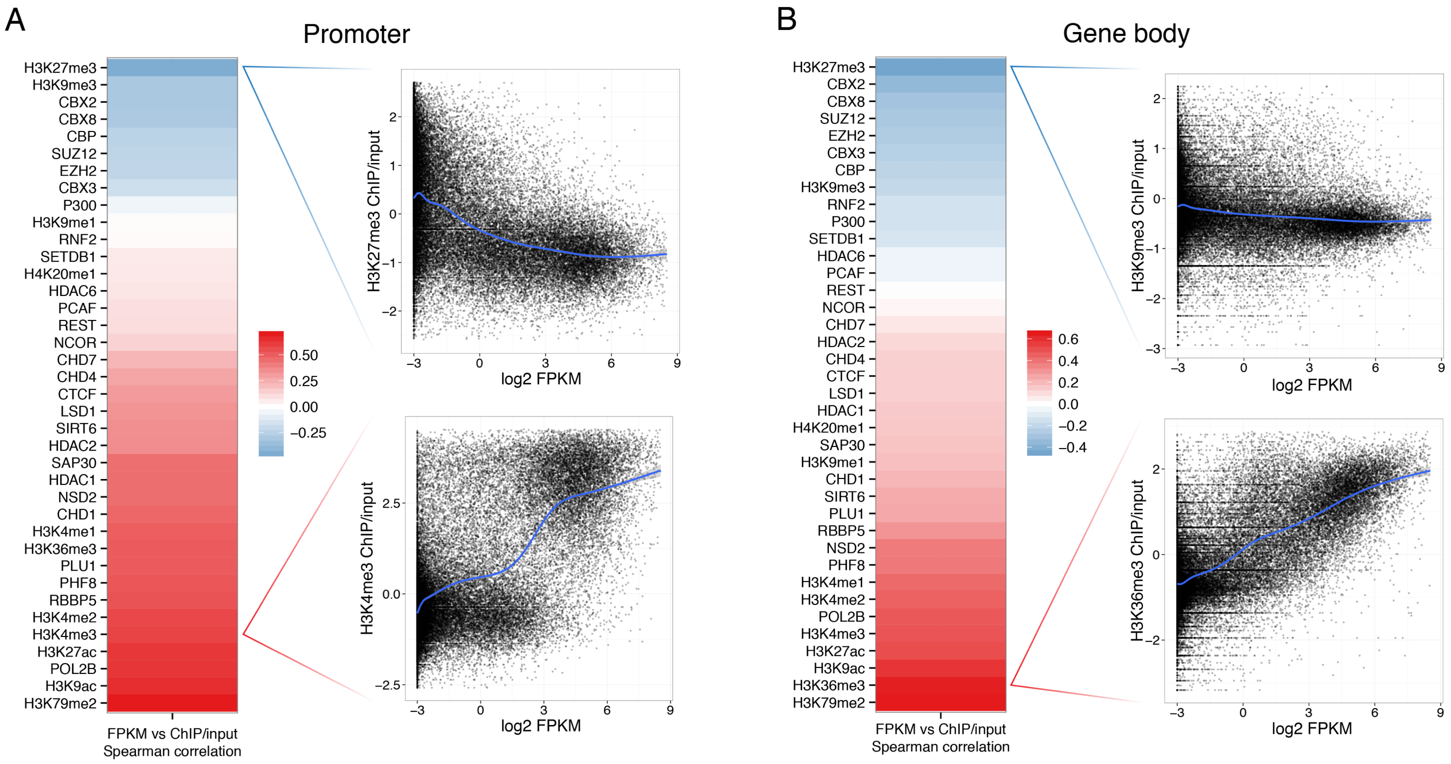

Figure S16: Chromatin marks correlate with gene abundance.
Over all genes, we computed the Spearman correlation between input FPKM and ChIP-Seq/input in the (A) promoter and (B) gene body, revealing known correlations between chromatin marks and gene abundance.

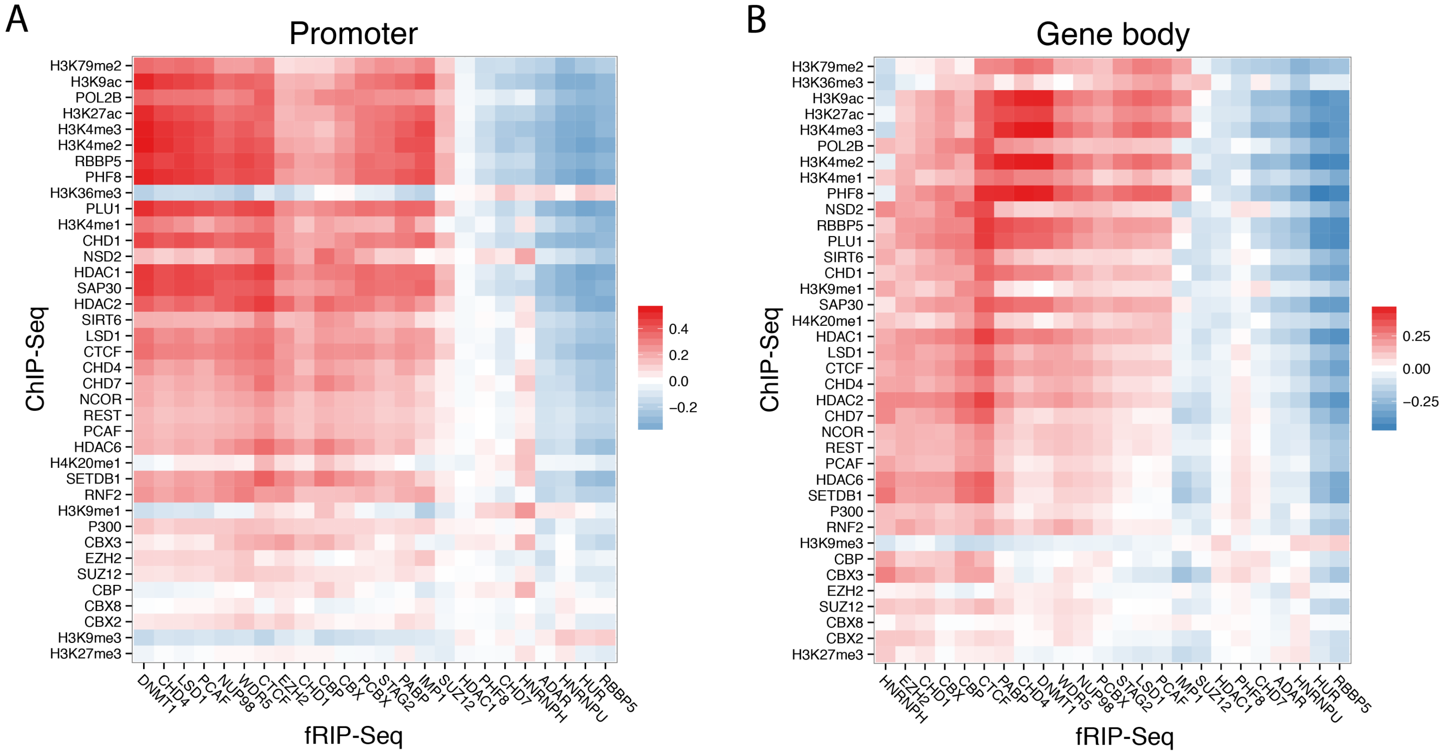

Figure S17: fRIP versus chromatin mark correlations are FPKM-driven.
Over all genes, we computed the Spearman correlation between each fRIP-Seq/input and each ChIP-Seq/input in the (A) promoter and (B) gene body. Because both fRIP-Seq/input and ChIP-Seq/input correlate with FPKM, these correlations are FPKM-driven and challenging to interpret.

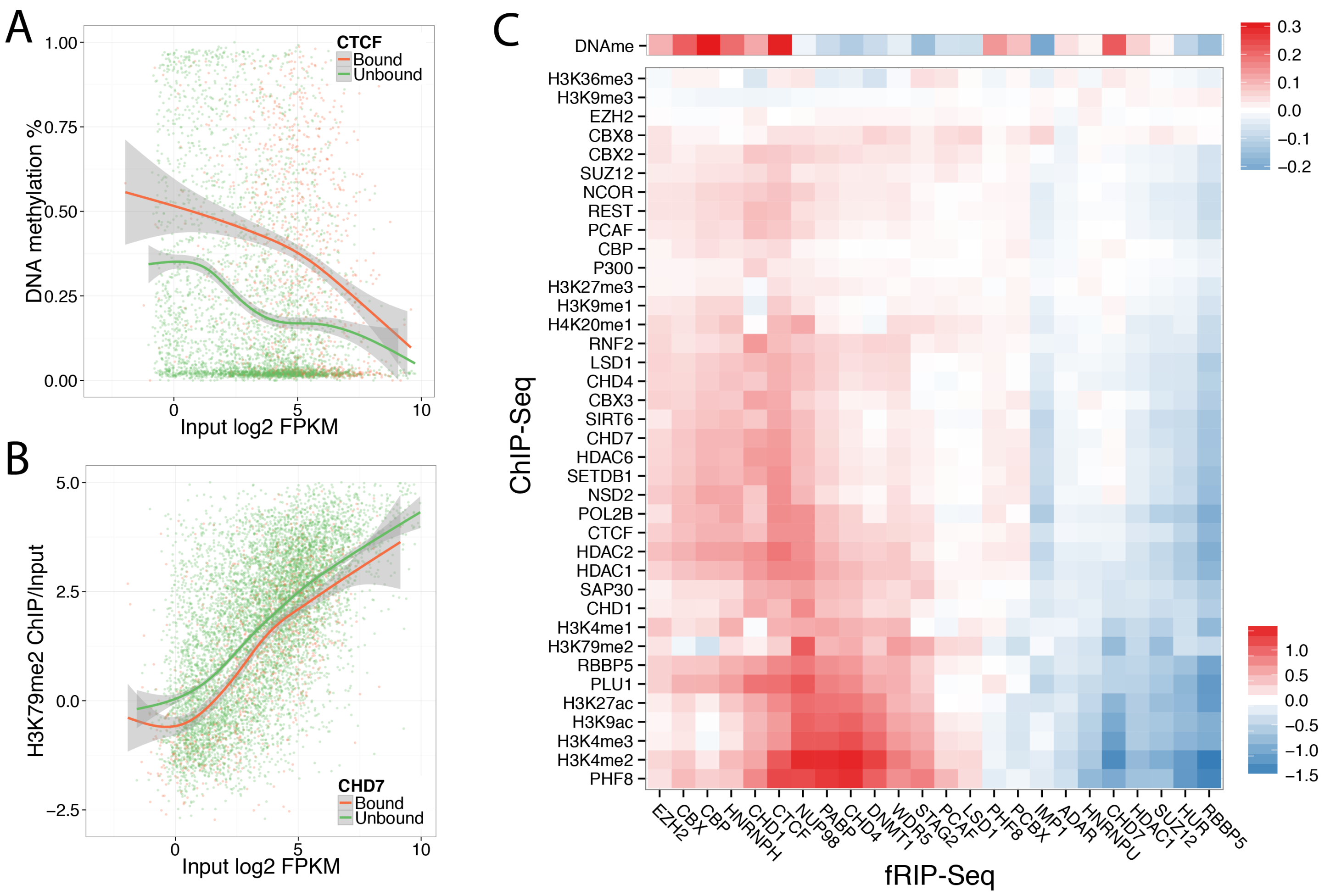

Figure S18: Protein binding to RNA relates to local chromatin over the gene body.

Because chromatin marks measured by RRBS (DNA methylation) or ChIP-Seq (histone modifications and modifiers) correlate with gene abundance (Figure 6 - figure supplement 2), we plotted this relationship separately for genes bound and unbound by each protein. (A) CTCF-bound RNAs have more DNA methylation over the span of the gene body, shown as a scatter plot of every gene with a generalized additive model regression. (B) CHD7-bound RNAs have less H3K79me2 in over the gene body. (C) For each chromatin mark and protein, we computed the difference between the bound and unbound regression lines and plotted as a heat map (Methods), revealing a clear difference in the relationship of certain proteins to activating chromatin marks.
